# GeneBits: ultra-sensitive tumour-informed ctDNA monitoring of treatment response and relapse in cancer patients

**DOI:** 10.1186/s12967-025-06993-3

**Published:** 2025-08-27

**Authors:** Julian Broche, Olga Kelemen, Aishwarya Sekar, Leon Schütz, Francesc Muyas, Andrea Forschner, Christopher Schroeder, Stephan Ossowski

**Affiliations:** 1 Institute of Medical Genetics and Applied Genomics, University of Tübingen, Tübingen, Germany; 2 Institute for Bioinformatics and Medical Informatics (IBMI), University of Tübingen, Tübingen, Germany; 3 German Cancer Consortium (DKTK), partner site Tübingen, a partnership between DKFZ and University Hospital Tübingen, Tübingen, Germany; 4 Department of Dermatology, University Hospital Tübingen, Tübingen, Germany

**Keywords:** Cell-free DNA, Circulating tumour DNA, Liquid biopsy, Next-Generation-Sequencing, Unique molecular barcode, Ultra-deep sequencing, Treatment monitoring, Relapse detection, Molecular residual disease

## Abstract

**Background:**

Circulating tumour DNA (ctDNA) in liquid biopsies has emerged as a powerful biomarker in cancer patients. Its relative abundance in cell-free DNA serves as a proxy for the overall tumour burden. Here we present GeneBits, a method for cancer therapy monitoring and relapse detection. GeneBits employs tumour-informed enrichment panels targeting 20–100 somatic single-nucleotide variants (SNVs) in plasma-derived DNA, combined with ultra-deep sequencing and unique molecular barcoding. In conjunction with the newly developed computational method umiVar, GeneBits enables accurate detection of molecular residual disease and early relapse identification.

**Results:**

To assess the performance of GeneBits and umiVar, we conducted benchmarking experiments using three different commercial cell-free DNA reference standards. These standards were tested with targeted next-generation sequencing (NGS) workflows from both IDT and Twist, allowing us to evaluate the consistency and accuracy of our approach across different oligo-enrichment strategies. GeneBits achieved comparable depth of coverage across all target sites, demonstrating robust performance independent of the enrichment kit used. For duplex reads with ≥ 4x UMI-family size, umiVar achieved exceptionally low error rates, ranging from 7.4×10^-7^ to 7.5×10^-5^. Even when including mixed consensus reads (duplex & simplex), error rates remained low, between 6.1×10^-6^ and 9×10^-5^. Furthermore, umiVar enabled variant detection at a limit of detection as low as 0.0017%, with no false positive calls in mutation-free reference samples. In a reanalysed melanoma cohort, variant allele frequency kinetics closely mirrored imaging results, confirming the clinical relevance of our method.

**Conclusion:**

GeneBits and umiVar enable highly accurate therapy and relapse monitoring in plasma as well as identification of molecular residual disease within four weeks of tumour surgery or biopsy. By leveraging small, tumour-informed sequencing panels, GeneBits provides a targeted, cost-effective, and scalable approach for ctDNA-based cancer monitoring. The benchmarking experiments using multiple commercial cell-free DNA reference standards confirmed the high sensitivity and specificity of GeneBits and umiVar, making them valuable tools for precision oncology. UmiVar is available at https://github.com/imgag/umiVar.

**Supplementary Information:**

The online version contains supplementary material available at 10.1186/s12967-025-06993-3.

## Background

Liquid biopsies (LBs) are minimally invasive samples collected from various types of body fluids (e.g. blood, urine, saliva, cerebrospinal fluid), associated with minimal stress for the patient and used in diagnostics [1, 2]. Cell-free DNA (cfDNA), mainly from dying immune cells, erythrocyte progenitors, and tissues of the cardiovascular system, is found in healthy individuals’ blood [3]. In cancer, DNA from dying tumour cells is often shed into the circulatory system. Circulating tumour DNA (ctDNA) harbours the tumour-specific somatic alterations and represents a fraction of the overall cfDNA [4]. While cfDNA shows a ladder-like size distribution with a peak at 167 bp, reflecting two windings around a nucleosome and flanking linker DNA, ctDNA is typically shorter in size (100–150 bp) [5, 6]. The half-life of cfDNA in blood is 0.25–2 h due to consistent clearance by kidneys, liver and spleen [7, 8]. This makes ctDNA a promising cancer biomarker, as it reflects the current ctDNA release into the bloodstream. Studies show a strong correlation between the relative ctDNA abundance and tumour burden measured by imaging technologies, supporting treatment decisions or relapse detection [9–11]. The presence of ctDNA after surgery or therapy is termed molecular residual disease (MRD) [12]. Numerous studies have shown that MRD is a prognostic marker of disease recurrence and a predictive biomarker of response to subsequent therapy [13–18].

**Fig. 1:**
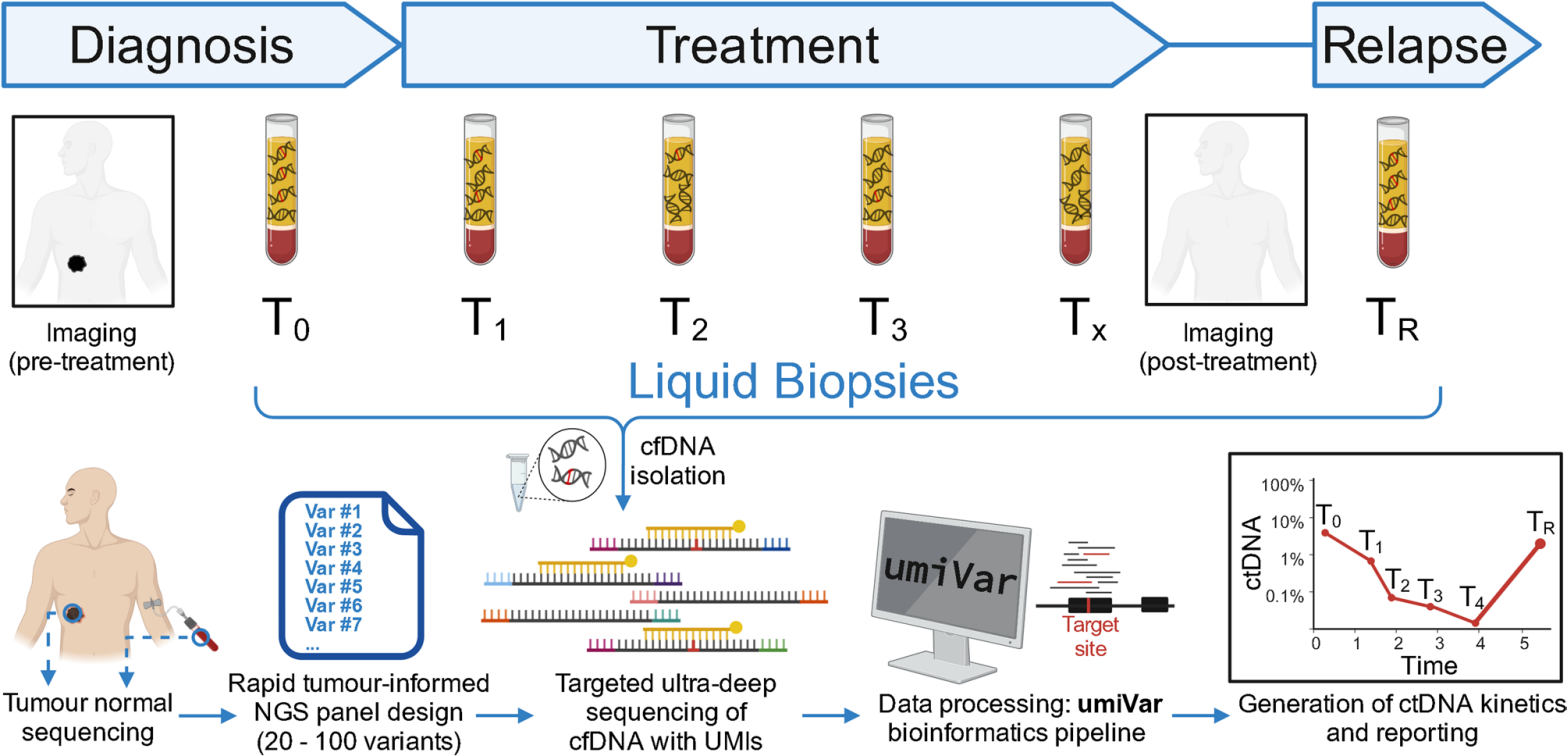
GeneBits diagnostics workflow for cfDNA-based cancer therapy monitoring. Exome-wide tumour normal sequencing is instructed from tumour biopsy and whole blood, later serving as reference for tumour-informed panel design. In parallel, a baseline (T_0_) liquid biopsy is collected before the start of therapy, during treatment (T_1_ – T_x_) and during follow-up care, for relapse monitoring (T_R_). Cell-free DNA (cfDNA) is isolated from plasma upon arrival and targeted Next-Generation Sequencing (NGS) with unique molecular identifiers (UMIs) is performed using the tumour-informed panel. NGS data are analysed with the umiVar bioinformatics software to calculate variant allele frequencies from circulating tumour DNA (ctDNA) in cfDNA. CtDNA kinetics over multiple collection timepoints are generated, serving as proxy for the patient’s tumour load. Molecular residual disease (MRD) is computed at all timepoints. Imaging is performed before and after treatment

A major challenge in ctDNA diagnostics is the low tumour allele fraction observed in cfDNA in early-stage cancers or tumours with weak blood supply, typically falling between 0.01 and 1% (Fig. 1) [19]. Various approaches have been developed to distinguish ctDNA from non-malignant cfDNA background, based on genomic or epigenomic features. For instance, ctDNA fragments can be identified based on cancer type-specific DNA methylation patterns [20, 21]. Moreover, somatic alterations like single-nucleotide variants (SNVs), insertions/deletions (indels), rearrangements and fusions serve as biomarkers for ctDNA quantification [22, 23]. Detecting somatic variants in patients with low tumour burden requires highly sensitive technologies, primarily droplet digital PCR (ddPCR) and Next-Generation Sequencing (NGS). Both methods allow to detect variant allele frequencies (VAFs) down to approximately 0.1%, which is in the range of error rates occurring during high-throughput sequencing [24]. Major constraints of ddPCR are the limited number of target regions per testing, uncertainty about PCR errors, unspecific fluorescent probe binding and off-target binding [25]. NGS technologies are mostly amplicon- or hybridization capture-based, with the latter offering better coverage uniformity, flexibility in target selection, analytical sensitivity and library complexity [26]. Current NGS-based methods include cancer-gene panels covering up to 250 genes, smaller hotspot-panels and tumour-informed panel designs featuring only SNVs previously detected in patient tumours [18, 27, 28]. The latter has since been employed by a growing number of studies [29–32].

We recently published the use of tumour-informed liquid biopsy for monitoring treatment in 84 melanoma patients using up to 30 patient-specific mutations [9]. We now generalise this approach, termed GeneBits, to support highly accurate tracking of ctDNA variants over time. We present a complete workflow for selection of monitoring variants, rapid panel design, library preparation, unique molecular barcoding, sequencing, and computational analysis of cell-free DNA from cancer patients in treatment or adjuvant setting. The performance of GeneBits was benchmarked with commercial cfDNA reference standards with known VAFs to evaluate the sensitivity and specificity of the workflow. The UMI-based multi-SNV error model of our novel tool umiVar enables error rates close to 1/million cfDNA fragments. Our statistical models combining up to 100 monitored variants enable accurate tracking of ctDNA levels over time, and the detection of molecular residual disease.

## Methods

### GeneBits workflow

**Fig. 2:**
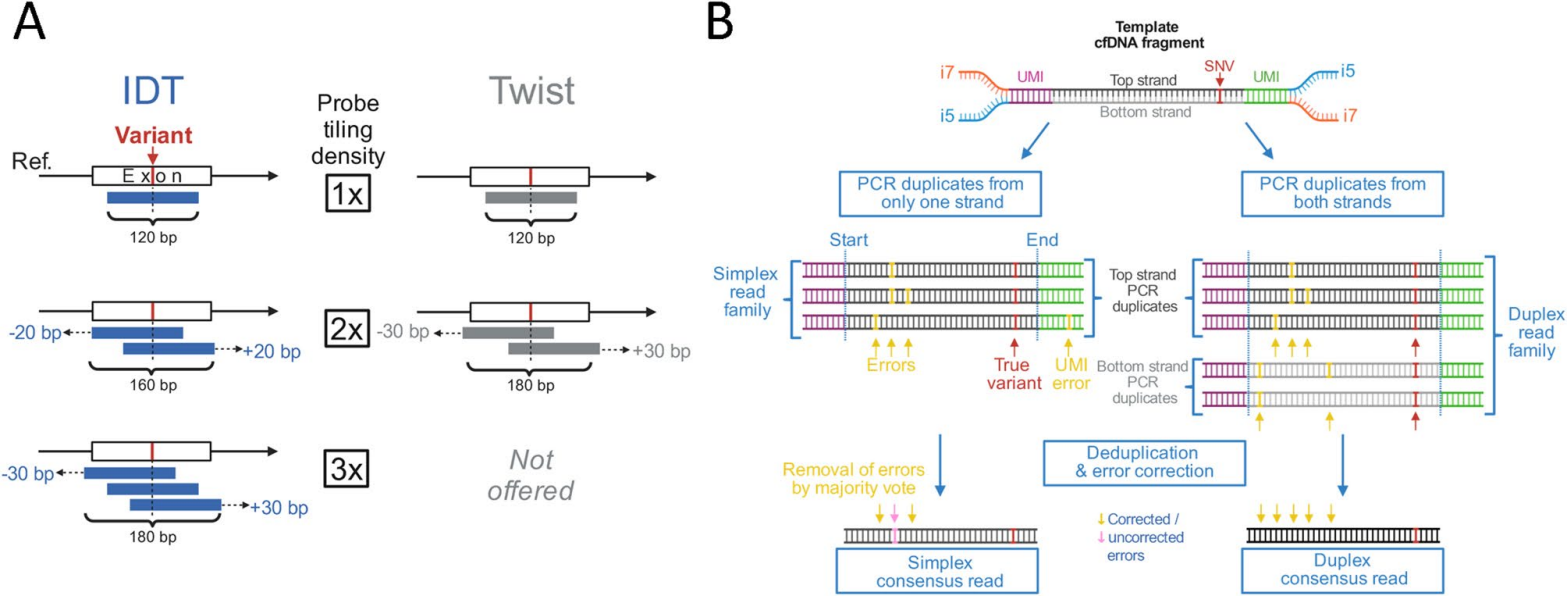
Enrichment and analysis settings for variant detection in liquid biopsies.** (A) **Probe design: We tested rapid-design hybridization-capture probes for target enrichment from two vendors: IDT and Twist. 120-bp probes are available in different tiling densities around the target site and vary in positioning. **(B)** UmiVar error correction: PCR duplicates as well as forward and reverse strand (duplex sequences) originating from the same DNA molecule are used to generate a consensus read sequence with very low error rates. A template cfDNA molecule with one real single-nucleotide variant (SNV) is flanked by two UMI sequences and Illumina i7 and i5 sequencing adapters. DNA fragments are amplified and duplicates are grouped into read families based on their fragment endpoints (start and end of alignments to the reference genome) and matching UMI sequences. The positioning of both UMIs relative to the i5 and i7 indexes is used to determine the strand of the template cfDNA molecule (dark grey = forward strand, light grey = reverse strand). A specified maximum number of errors in UMI sequences is allowed for UMI grouping. Read families are collapsed into consensus reads and errors are removed by majority vote. Simplex consensus (SC) calling (left workflow) generates one family of reads from only one strand, if no read from the opposite strand is present, and collapses those reads into a strand-specific SC read. Duplex consensus (DC) calling (right workflow) groups together the reads derived from both strands of the same template cfDNA molecule and collapses them into a duplex consensus read. In umiVar default mode, SC and DC reads are used together for variant calling, while considering different error models of SC and DC

GeneBits uses a tumour-informed approach for monitoring somatic variants in cell-free DNA from cancer patients. Liquid biopsies are collected at therapy start (T_0_, baseline), every 2–6 weeks during treatment (T_1_ – T_x_) and during follow-up care for relapse detection (T_R_) (Fig. 2). CfDNA is then isolated from plasma. Whole-exome sequencing (WES) of tumour and normal samples is conducted for molecular profiling and somatic variant calling, followed by selecting 20–100 SNVs for rapid panel design. Library preparation is performed with the hybridization capture NGS workflow from either IDT or Twist, both allowing the ligation of unique molecular identifiers (UMIs) to template cfDNA molecules. Target enrichment utilizes the tumour-informed panels and libraries are sequenced at ultra-high depth. NGS data are processed with the umiVar bioinformatics pipeline, consisting of methods for UMI-based barcode correction, variant calling and detection of molecular residual disease (MRD). UmiVar and comprehensive documentation are available at https://github.com/imgag/umiVar and additional details are provided in the supplementary information. Variant allele frequencies (VAFs) of somatic variants are calculated for multiple timepoints, and VAF kinetics are reported to the clinicians. The entire workflow can be executed within 3–4 weeks, providing first datapoints for the evaluation of treatment response at a clinically relevant time point (Suppl. Figure 1).

## Tumour-informed NGS panel design

Tumour normal sequencing is performed following Eckard et al. 2023 [33]. In brief, tumour DNA is extracted from FFPE tissue blocks, and normal DNA from whole blood using standard protocols. Input consists of 10–100 ng FFPE DNA and 50–100 ng of high-molecular weight DNA from blood. Pre-capture libraries are prepared using the Twist Library Preparation EF Kit 2.0 (Twist Bioscience). Target enrichment uses either a whole-exome or comprehensive cancer panel, with the Twist Standard Hybridization Reagent Kit v2 (Twist Bioscience) according to the manufacturer’s protocol (version 2). Libraries are sequenced on Illumina NovaSeq in paired-end mode (2 × 150 bp), achieving 400 million reads for tumour and 140 million for normal samples. The megSAP pipeline (https://github.com/imgag/megSAP, https://github.com/imgag/ngs-bits) processes tumour-normal pairs to generate a list of somatic variants. NGS data are imported into the clinical decision support system GSvar, providing an integrated ctDNA panel design tool (https://github.com/imgag/ngs-bits/blob/master/doc/GSvar/index.md).

## Selection of monitoring variants for rapid tumour-informed panel design

**Fig. 3:**
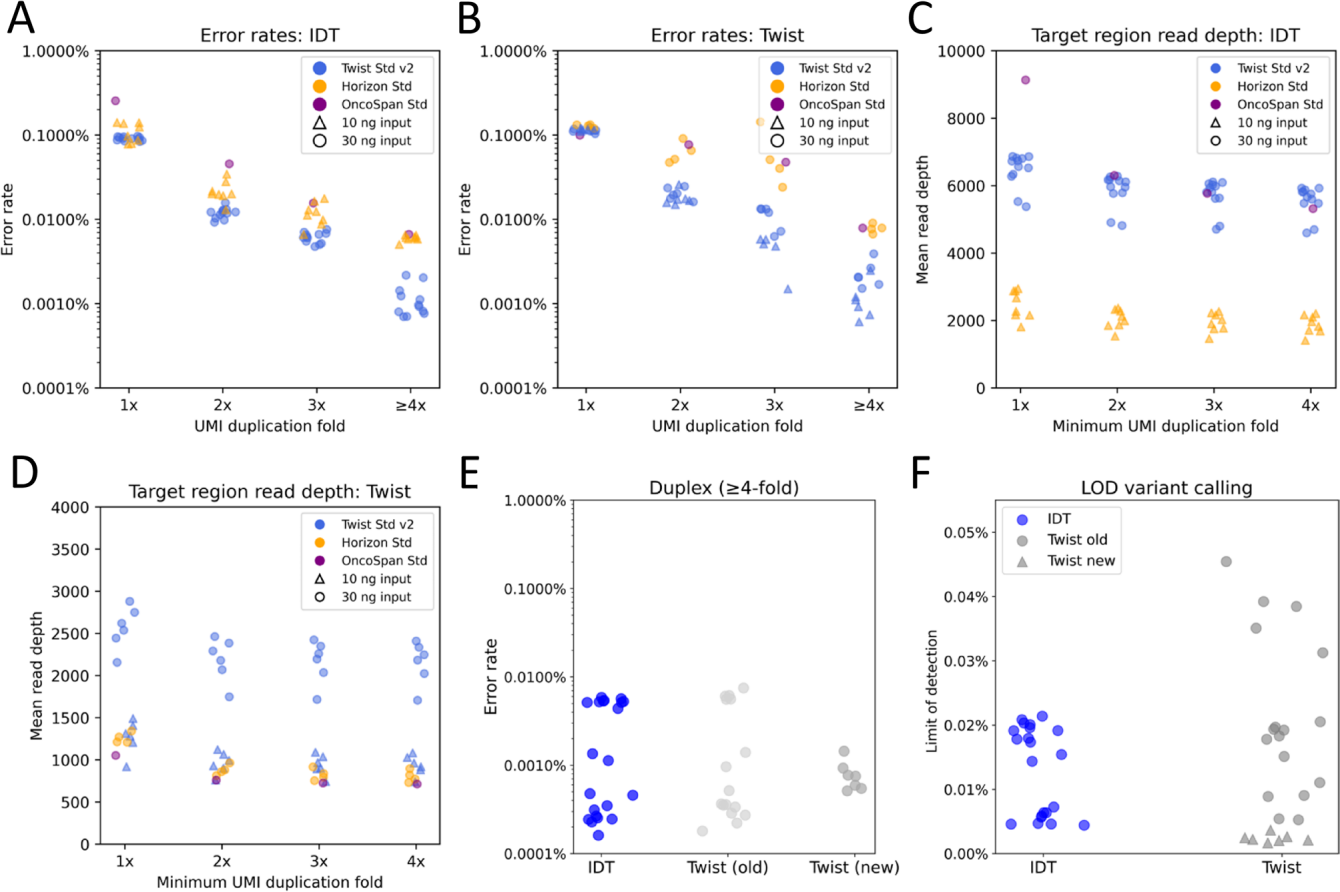
Coverage and error metrics after UMI-assisted consensus calling. Deduplicated read depths and error rates for different commercial cell-free DNA reference standards (Twist Std v2, Horizon Std, OncoSpan Std), tested with the IDT and Twist library preparation and hybridization capture workflows. **(A, B)** Error rates when using only consensus reads formed by 1x, 2x, 3x, or ≥ 4x UMI families (PCR duplicates) using the IDT (A) or Twist (B) workflows. **(C, D)** Depth of coverage when using only consensus reads formed by ≥ 1x, ≥ 2x, ≥ 3x, or ≥ 4x UMI-families (PCR duplicates) using the IDT (C) or Twist (D) workflows. **(E)** Comparing error rates between three workflows using only duplex-consensus reads formed by ≥ 4x UMI-families. **(F)** Comparing the limit of detection (LOD) between three workflows using only duplex-consensus reads formed by ≥ 4x UMI-families. (Lower is better for error rates and LOD, higher is better for depth of coverage)

For designing tumour-informed NGS panels, typically 20–100 somatic single-nucleotide variants (SNVs) and short indels are selected. The selection process follows criteria to minimize technical artefacts. Exonic variants are prioritized, while intronic or intergenic (e.g. *TERT* promoter) variants may be included. Variants near repetitive elements or in low complexity regions are excluded, and clustered variants and nearby SNPs are avoided. NGS panels contain tumour-specific driver and passenger mutations to reflect the tumour’s unique mutational landscape, but may also include resistance hotspots, fusion sites of structural variants, or other targets. Fingerprint SNPs can be included as internal control. The synthesis of 120-bp biotinylated oligonucleotide probes is performed by IDT or Twist. Hybridization capture probes were available in 1x, 2x (IDT, Twist) or 3x (IDT) tiling densities (Fig. 3A). The exact positioning of the hybridization sites relative to the target slightly varied between both vendors. Panel designs are submitted to IDT via the online panel design and ordering tool (https://eu.idtdna.com/pages/products/next-generation-sequencing/methods/minimal-residual-disease-(mrd)-research), or to Twist by standard customer support.

## Library Preparation and sequencing

GeneBits’ performance was benchmarked using three different kits for library preparation and oligo-enrichment provided by IDT and Twist. Benchmarking experiments used 10 ng, 15 ng or 30 ng of cfDNA reference standard as input, while patient samples contained 14–60 ng cfDNA.

The xGen cfDNA & FFPE DNA Library Prep Kit (IDT, cat. #10006203) was first benchmarked. Upon end-repair of cfDNA, two UMI adapters containing a fixed 8-bp sequence from a pool of 32 UMIs were ligated to the template molecules. 8-bp unique dual indexes (UDIs) were introduced during pre-capture PCR (9–10 cycles) using xGen UDI Primers (IDT, cat. #10005922) and KAPA HiFi HotStart ReadyMix (Roche Diagnostics, cat. #07958927001). Quality control was conducted on Agilent 4200 TapeStation using the D1000 ScreenTape (Agilent Technologies, cat. #5067–5582), and on a Qubit 4 fluorometer using the dsDNA BR Assay-Kit (Invitrogen, cat. # Q32850). Hybridization capture was carried out for 16 h at 65 °C with xGen Universal Blockers for TruSeq libraries (IDT, cat. #1075474), and Human Cot DNA provided in the xGen Hybridization and Wash Kit v2 (IDT, cat. #10010351/#10010353"10010351/#10010353"/>). Libraries were typically pooled in 4- to 6-plexes (500 ng/library). In each hybrid capture reaction, 2 µl of IDT Discovery Pool (tumour-informed panel, updated name: xGen MRD Hyb Panels) and 2 µl of xGen Human ID Research Panel v1.0 (IDT, Cat. #1075703) were used. KAPA HiFi HotStart ReadyMix and the xGen Library Amplification Primer Mix (IDT, cat. #1077676) was used for post-capture PCR (12 cycles). Final quality and concentrations were assessed by Agilent 4200 TapeStation using the High Sensitivity D1000 ScreenTape assay (Agilent Technologies, cat. # 5067–5584), and with the Qubit dsDNA HS Assay Kit (Invitrogen, cat. # Q33230).

Two versions of the Twist pre-capture workflows were employed for benchmarking. The Twist Library Preparation MF Kit (Twist Bioscience, cat. # 104176) referred to as “Twist” or “Twist (old)” was used for most analyses. An updated version of the workflow was provided by Twist recently, which at the time was still in alpha-testing. It is currently available as Twist cfDNA Library Preparation Kit (Twist Bioscience, cat. #107604) and hereinafter referred to as “Twist (new)”. For the Twist workflows, pre-capture libraries were prepared using 10–30 ng cfDNA reference standard. 5-bp randomized UMI adapters from the Twist UMI Adapter System (Twist Bioscience, cat. #105040) were ligated to DNA fragments. 10-bp UDIs were introduced during library amplification (8–9 cycles) using Twist UDI primers (Twist Bioscience, cat. #105093) and Equinox Library Amp Mix (Twist, cat. #104107). Quality was assessed on Agilent 4200 TapeStation using the D1000 ScreenTape assay (Agilent Technologies, cat. # 5067–5582) and concentration measured via Qubit dsDNA BR Assay-Kit (Invitrogen). Libraries were pooled in 4- to 6-plexes, using 250–500 ng (Twist (old)) or 1000 ng (Twist (new)) per library. Hybridization capture was performed according to Twist Target Enrichment Standard Hybridization v2 protocol, using the Twist Target Standard Hyb and Wash v2 kit (Twist, cat. #104446). After post-capture PCR (15 cycles) with Equinox Library Amp Mix, the quality and concentration of library pools was controlled by D1000 ScreenTape assay and Qubit dsDNA BR Assay.

Library preparation and hybrid capture of reference standards and patient cell-free DNA samples were performed as per the protocols of Integrated DNA technologies (IDT) and Twist Biosciences. Library pools were sequenced on Illumina NovaSeq6000 and NovaSeq X Plus (Illumina, Inc.) on patterned SP, S1, S2 and 1.5B flowcells. Sequencing was performed in paired-end mode (2 × 100 bp), aiming to obtain ~ 100,000x raw read depth on target regions for each library.

## Cell-free DNA reference standards for benchmarking

For the evaluation of GeneBits and umiVar, three different commercial cfDNA reference standards with varying VAFs were employed: (I) OncoSpan cell-free DNA reference standard (“OncoSpan Std”) (Horizon Discovery Ltd., cat. #HD833), containing 386 variants across 152 cancer-related genes with expected VAFs between 1% and 100%. (II) Horizon Multiplex I cell-free DNA Reference Standard Set (“Horizon Std”) (Horizon Discovery Ltd., cat. #HD780), containing different dilutions of oncogenic variants (5%, 1%, 0.1%) and a wild-type control with expected VAFs of 0%. (III) Twist cfDNA Pan-Cancer Reference Standard v2 (“Twist Std v2”) (Twist Bioscience, cat. #104549), encompassing more than 300 variants in dilutions of 5%, 2%, 1%, 0.5%, 0.25%, 0.1% and 0%.

From OncoSpan Std, 30 variants (23 SNVs, 7 indels) with expected VAFs ranging from 1 to 93% were selected for panel design (Suppl. Table 1 A). Horizon Std contains eight variants (six SNVs, two indels), all included in the benchmark analysis (Suppl. Table 1B). From the Twist Std v2, 50 cancer-associated SNVs were included into the panels (Suppl. Table 1 C). IDT panels were designed individually for each cell-free DNA standard (Suppl. Table 1 A-C) and 120-bp probes were synthesized for each target region. For the Horizon Std and the OncoSpan Std, a small set of fingerprint SNPs (*n* = 14) was included as internal quality control. For Twist Custom Panels (Twist Biosciences, cat. #101001), 120-bp probes were ordered in 2x-tiling density. A combined panel was designed, which included all tested reference standards (Suppl. Table 1D), 14 fingerprint SNPs, and all 76 fingerprint SNPs found in the xGen Human ID Research Panel v1.0 (IDT, cat. #1075703). For the updated Twist workflow, a predesigned control panel was obtained from Twist, covering all variants included in Twist Std v2 (Suppl. Table 1E).

### UmiVar: consensus generation and error models

The detection of ultra-low frequency variants with high specificity is challenging due to errors introduced during DNA extraction, PCR and sequencing. To generate accurate consensus sequences umiVar uses UMIs, fragment endpoints and read orientations to identify PCR duplicates (Fig. 3B). Read pairs with opposite orientation but identical fragment endpoints and containing reciprocal UMIs are considered as originating from the different strands of the same DNA molecule (termed duplex reads). Grouping of duplicates by UMI sequences tolerates sequencing errors in the UMIs using an adjustable edit distance (typically 1–3 mismatches depending on the combined length of up- and downstream UMIs).

All copies of one original DNA fragment (termed UMI-family) are used to generate a multiple sequence alignment with majority voting for each nucleotide. Positions with < 75% concordance are masked (nucleotide replaced by ‘N’). For high concordance positions, a joint quality value is computed as the mean of base qualities. Reads with highly discordant positions (user-parameter) are filtered out. Consensus reads (‘deduplicated reads’) with updated base qualities are returned in BAM format. To facilitate computation of elaborate error models for subsequent variant calling we distinguish several quality levels for consensus reads. First, reads are categorized into four UMI-family sizes (1, 2, 3, and ≥ 4 duplicate reads from either strand). Second, we define a simplex consensus (SC, BAM-tag YD = 0) if only reads from one strand were available and a duplex consensus (DC, BAM-tag YD = 1) if the UMI-family contains reads from both strands. Combining both SC and DC in one BAM file is termed mixed consensus (MC). Alternatively, SC reads can be filtered to further reduce error rates using only DC.

## Variant calling and detection of molecular residual disease

UmiVar computes separate error models for each consensus quality level (UMI-family sizes 1x, 2x, 3x and ≥ 4x in MC mode), and optionally for each possible nucleotide change (A-C, A-G, A-T, C-G, C-T, G-T). Pileup files are generated for each UMI-family size with observed nucleotides and base qualities per covered genomic position. Background error rates are calculated for each pileup file considering all covered positions on autosomes, while excluding known germline variants, monitored somatic variants or possible variants with allele frequency (AF) ≥ 20%. Optionally, background error rates for the six nucleotide-change groups can also be separated.

For variant calling, Fisher’s Exact Test calculates *p*-values for each position indicating the significance of an alternative call (i.e. somatic variant) based on the combined nucleotide observations from all pileup files. Pileup files from smaller UMI-families (1x, 2x and 3x) can be excluded. To compute the *p*-value the AF of a potential variant is compared to the expected background noise using the appropriate error model for each group. A proportion Z statistics and fold change between potential variants and background errors are computed, along with minimum detectable VAF based on the error models, representing the limit of detection (LOD). Monitoring variants count as called if supported by at least three consensus reads and at a *p*-value ≤ 0.05.

If GeneBits is used to monitor known (tumour-informed) somatic mutations, umiVar calculates the MRD significance by first excluding monitored variants whose VAF deviates by more than three standard deviations from the average VAF across all monitored sites. It then aggregates mutant and wildtype reads from all target loci and compares their ratio to the same ratio in reads from the ± 60 bp flanking regions using a single contingency table evaluated by Fisher’s Exact Test. UmiVar features a second, de novo variant calling mode, using a combined negative beta-binomial error model for all UMI-family sizes. However, this feature, mainly utilized for detection of novel resistance mutations, was not applied and benchmarked in this study. Details can be found on umiVar’s GitHub page.

## Integration in the clinical decision support system GSvar and longitudinal monitoring

Variant selection, panel design and data analysis are seamlessly integrated in our clinical decision support system megSAP (https://github.com/imgag/megSAP) and its graphical user interface GSvar (https://github.com/imgag/ngs-bits/tree/master/doc/GSvar) [34]. GSvar generates BED files of regions of interest, e.g. selected monitoring variants, usable for panel ordering at IDT or Twist. Apart from a stand-alone mode, the NGS analysis of umiVar can also be performed as part of megSAP, allowing the inclusion of results in the clinical database of megSAP (NGSD) and visualisation in GSvar. Additional python scripts facilitate the plotting of VAF kinetics of a single patient over multiple sampling time points with significance values and MRD.

### Comparison of computational pipelines

The performance of the umiVar bioinformatics pipeline was compared to IDT and Twist workflows, which use various open-source tools like Picard (https://broadinstitute.github.io/picard/) and fgbio (http://fulcrumgenomics.github.io/fgbio/) to process raw sequencing data and generate consensus reads (Suppl. Figure 2). FASTQ files from the 0.1% dilution samples of Twist Std v2 were used for this comparison. The IDT-processed library was analysed using both the umiVar and IDT bioinformatics pipelines, while the Twist-processed library was analysed with both the umiVar and Twist pipelines. For reasons of comparability, simplex and duplex consensus generation were performed separately. An edit distance of two was used for Twist libraries and three for IDT libraries within their combined UMI sequence. The Twist pipeline ended after consensus calling, while IDT included VarDict (https://github.com/AstraZeneca-NGS/VarDict) for variant calling. In addition, we tested the VarScan2 (https://github.com/Jeltje/varscan2) variant caller, with the minimum VAF threshold set to zero. For clarity and direct comparability with umiVar, the performance of consensus generation and error correction of the vendor workflows was evaluated with the umiVar variant caller, using consensus BAM files as input. The coverage depth at target sites was calculated for UMI-family size of ≥ 4.

## Results

With GeneBits we introduce a novel diagnostic workflow for monitoring somatic variants in ctDNA from liquid biopsies. The process begins with broad genomic profiling of tumour tissue through tumour normal sequencing (e.g. exome sequencing), followed by the selection of 20–100 somatic SNVs for panel design (Fig. 2). Liquid biopsies are collected at therapy start (baseline sample, T_0_), during treatment (T_1_ – T_x_), and follow-up (T_R_) in special blood collection tubes. After plasma separation and cell-free DNA isolation, sequencing libraries are prepared with unique molecular identifiers (UMIs) attached to template molecules. The tumour-informed panels are used for target enrichment, and libraries are sequenced to ultra-high depth (~ 100.000x raw coverage). Sequencing data are processed using the umiVar software for UMI and duplex-read assisted error correction and variant calling. An error model considering nucleotide-change, PCR-duplicate, simplex and duplex specific error rates enables LODs as low as 0.0017% due to error rates of 7.4 × 10-7 in consensus sequences. Panel-wide variant allele frequencies (VAFs) serve as tumour burden proxy and are combined to compute molecular residual disease (MRD) probabilities. Longitudinal sampling generates VAF kinetics, providing insights into therapy response or relapse emergence. GeneBits enhances clinical decision-making by adding temporal dimension to imaging techniques.

UmiVar, an essential part of GeneBits, integrates novel statistical methods for analysing hybridization capture-based NGS data with UMIs (see Methods). Here we benchmark umiVar’s performance using two UMI-based NGS protocols supported by GeneBits from Integrated DNA Technologies (IDT) and Twist Bioscience, featuring 8-bp fixed UMIs or 5-bp randomized UMIs respectively. We also included limited evaluation of a new Twist workflow version released during manuscript preparation. For benchmarking, we used three cell-free DNA reference standards (see Methods): the Twist cfDNA Pan-cancer reference standard kit v2 (“Twist Std v2”), the Horizon Multiplex I cfDNA Reference Standard Set (“Horizon Std”), and the OncoSpan cfDNA reference standard (“OncoSpan Std”). These commercial cfDNA standards supply limitless, well-characterized material with vendor-defined VAFs and clinically relevant mutations that mimic native cfDNA, enabling reproducible benchmarking studies for realistic allele-frequency ranges. In all experiments, 10–30 ng input material was used, reflecting typical yield from 2 to 5 ml plasma samples.

### Benchmarking target enrichment efficiency of GeneBits

The umiVar bioinformatics pipeline generates single-strand consensus (termed simplex consensus, SC) reads and, when at least one read from the opposite strand is available, produces duplex consensus (DC) reads (Fig. 3B). The error-corrected consensus sequence is calculated using PCR duplicates identified by UMI barcodes and read alignment position (see Methods). Together, simplex and duplex consensus reads form mixed consensus (MC), which are by default used for variant calling. Deduplicated read coverage was calculated for IDT and Twist libraries using the integrated umiVar variant caller.

**Fig. 4:**
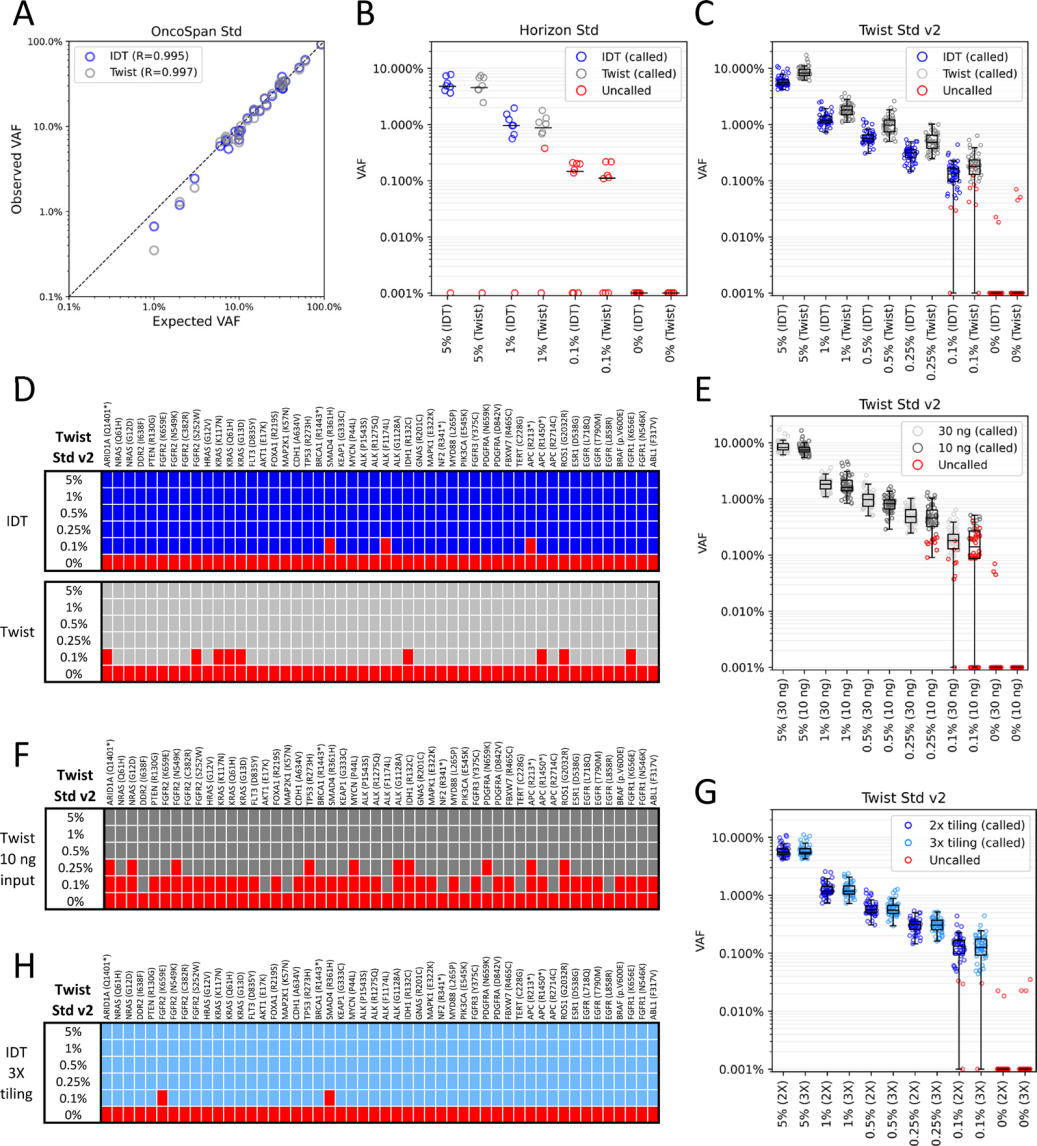
Sensitive detection of low and ultra-low variant allele frequencies (VAF) with umiVar. Libraries for cell-free DNA reference standards from Horizon and Twist Std v2 were prepared with targeted NGS workflows from IDT (blue) and Twist (gray), using 2X density for hybridization-capture probes by default. Data were processed with the umiVar pipeline. Data are shown for ≥ 4x UMI-families. Blue = variant called from IDT workflow, gray = variant called from Twist workflow, red = uncalled variant (< 3variant reads, and/or p-value >0.05). **(A)** 30 variants from the OncoSpan Std enriched with the IDT (10 ng input) and Twist (30 ng input) workflows. Plotted are expected VAFs (validated by the vendor) vs. observed VAFs and the Pearson correlation coefficient (“R”). **(B)** Horizon Std (5%, 1%, 0.1%, 0% dilutions) prepared with IDT (10 ng input) and Twist (30 ng input) workflows, black horizontal lines represent the median VAF. **(C)** Twist Std v2 (5%, 1%, 0.5%, 0.25%, 0.1%, and 0% dilutions) prepared with IDT and Twist workflows (30 ng input). **(D)** Detection of individual variants of the Twist Std v2 using the IDT and Twist workflows (30 ng input). **(E)** Comparing variant detection efficiency for 30 vs. 10 ng input of Twist Std v2, prepared with the Twist workflow. **(F)** Detection of individual variants of the Twist Std v2 using only 10 ng as input for the Twist workflow. **(G)** Comparing variant detection efficiency for 2x vs. 3x tiling density of hybridisation-capture probes for the Twist Std v2 (30 ng input) using the IDT workflow. **(H)** Detection of individual variants of the Twist Std v2 (30 ng input) enriched with 3x tiling density in the IDT workflow. **(B/C/E/G)** Pseudo-count of 0.001% added to each variant for visual clarity

IDT libraries showed approximately twice the coverage at target sites compared to the Twist workflow, even with identical reference material input (Suppl. Figure 3 A, Fig. 4A and 4B). Increasing the DNA input from 10 ng to 30 ng improved coverage, yielding two- to three-fold higher target region read depths, as expected due to the higher number of unique molecules in the sample. IDT libraries produced more deduplicated reads relative to raw sequencing depth (Suppl. Figure 3 A). When normalized to input DNA amounts, IDT showed higher library complexity than both the old and new Twist workflows (Suppl. Figure 3B). With a mean coverage of 233.4 reads per ng DNA for IDT, assuming 1 ng represents 303 haploid genome equivalents, approximately 77% of template DNA molecules appeared in final NGS libraries (Suppl. Figure 3 C). Duplex consensus read fraction was similar across workflows: 53% for IDT, 46% for old and 56% for new Twist (Suppl. Figure 3D). However, IDT achieved the highest duplex read depth relative to raw sequencing input (Suppl. Figure 3E), producing three times more duplex reads than the old Twist and 1.5 times more than the new Twist workflow (Suppl. Figure 3 C and 3 F).

### Benchmarking UmiVar error correction model

Conceptually, umiVar uses PCR duplicates for correcting PCR and sequencing errors. Reads without a PCR copy (“1x”)—and therefore not subjected to the correction step— exhibited a median error rate of 0.093% (0.08–0.25%) for IDT, 0.116% (0.10–0.13%) for the old Twist workflow, and 0.208% (0.20–0.21%) for the new Twist workflow (Fig. 4A and B). Error correction using a single PCR copy (“2x”) reduced median error rates by 7.2-fold (IDT), 5.7-fold (Twist old) and 4.3-fold (Twist new). For MC reads with at least four PCR copies (“≥4x”), median error rates decreased further to 0.002% (0.0007–0.006%) for IDT, 0.0021% (0.0006–0.009%) for old Twist, and 0.0013% (0.001–0.0023%) for new Twist workflow, representing 46-, 56- and 163-fold reductions respectively, compared to reads without a PCR copy. Twist Std v2 consistently displayed lowest error rates across reference standards, while Horizon Std and OncoSpan Std showed approximately 3-fold higher rates, suggesting remaining errors in ≥ 4x UMI-families likely stem from base inclusion errors during reference standard synthesis.

Filtering out reads with no or low numbers of PCR copies substantially reduced background noise. Increasing the minimum required duplication level (termed minimum UMI-family size) from 1x to ≥ 4x had only a mild impact on coverage depth in MC reads, indicating that few reads lacked multiple PCR copies (Fig. 4C and D).

The umiVar variant calling module contains an optional post-filtering step solely using DC reads, which removes SC reads from the MC alignment file. Considering only duplex reads with minimum UMI family size of four reduces error rates to 0.00059% (0.000074–0.007%) (Fig. 4E) with 50% reduction in read depth (Suppl. Figure 3 C). Duplex consensus mode should therefore be chosen for highest precision applications, while mixed consensus mode provides better sensitivity at increased error rates beneficial for monitoring known tumour mutations.

The reduction in error rates in ≥ 4x UMI-families yields a very low limit of detection (LOD), defined by the threshold for detectable VAFs at significant level. The LOD across tested samples using MC mode had a median of 0.015% (0.0017–0.0455%), and was lowest for the new Twist workflow (Fig. 4F). The generation of MC reads using umiVar strongly improved error rates, which consequently lowers false positive variant detection probability. For this reason, the following analyses used MC mode with minimum UMI family size of four. In addition, umiVar applies quality filters requiring at least three DNA fragments with ≥ 4 UMI family size and *p*-value ≤ 0.05 (see Methods) to flag variants as real.

### Benchmarking detection of low-frequency variants

**Fig. 5:**
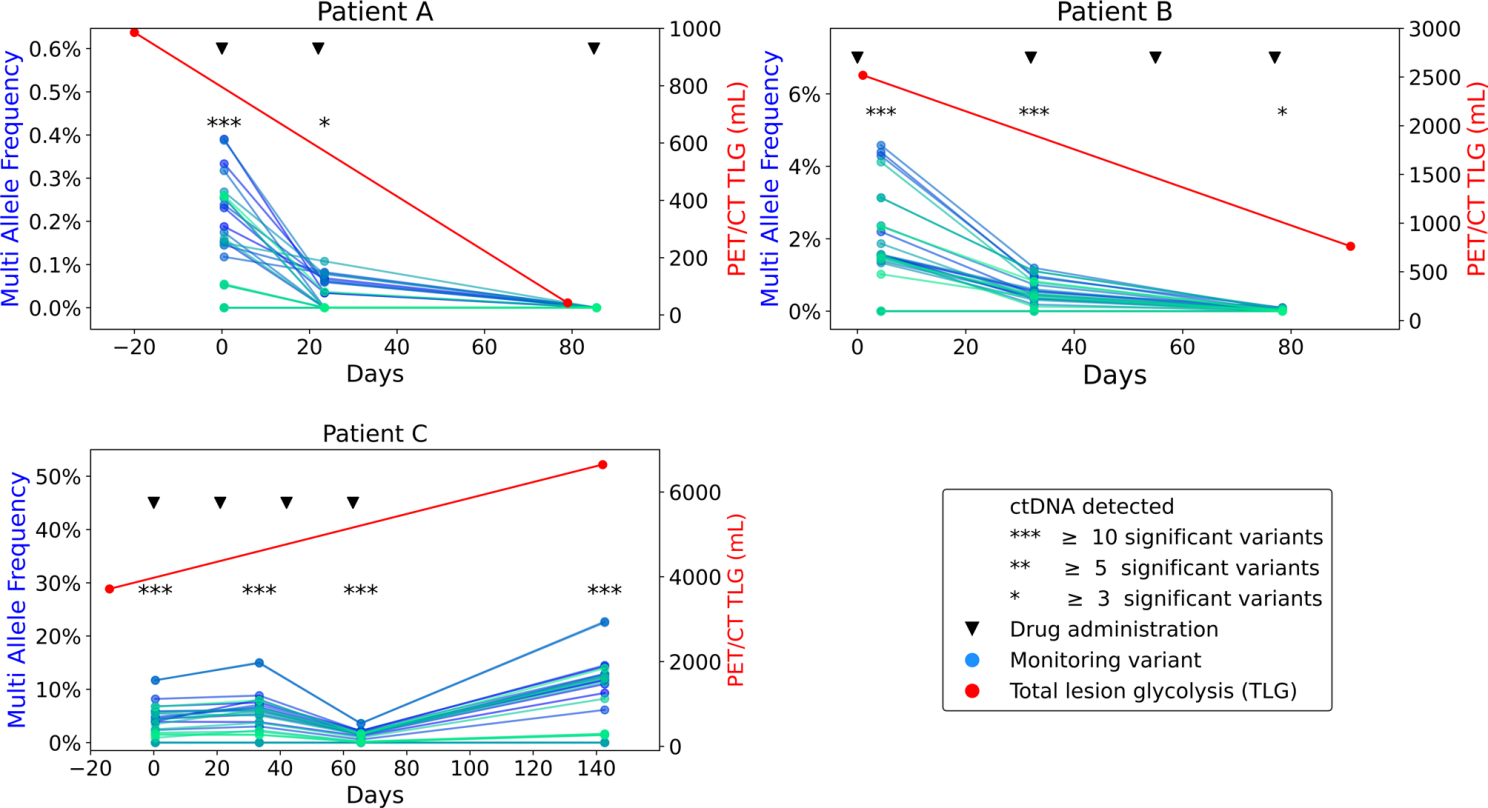
GeneBits used for monitoring melanoma patients under treatment and detection of MRD.Data obtained from PET/LIT study (PMID: 39384805).Reanalysis of three patients with the latest version of umiVar. Liquid biopsies were obtained from patients receiving Ipilimumab/Nivolumab treatment (▼). A tumour-informed panel containing 30 variants was designed for each patient. Variant allele frequencies (VAF) for all variants were calculated with umiVar for ≥ 2x UMI-families. VAF kinetics are displayed for each of the monitored variants (blue/turquoise dots). Total lesion glycolysis (TLG, red dots) was measured by PET/CT before the start (Day 0) and around the end of therapy. Asterisks indicate the number of significant (Fisher’s Exact test) variants per liquid biopsy. ctDNA = circulating tumour DNA. Patient A showed progress under treatment. MRD was detectable for patient B after 80 days of treatment. No MRD was detected for patient C after 85 days of treatment

CtDNA fractions in cell-free DNA can vary by multiple orders of magnitude, based on tumour localization, volume and the occurrence of metastasis. The OncoSpan Std contains over 380 variants across 152 cancer-related genes, covering a wide range of VAFs (1-100%). A custom panel was designed to enrich 23 SNVs and 7 indels, with expected VAFs between 1% and 92.5%, reflecting a medium to high tumour burden in a patient. Using GeneBits, all 30 variants were detected in IDT and Twist workflows. The observed VAFs correlated highly (*R* = 0.995 and *R* = 0.997, respectively) with expected VAFs confirmed via NGS and ddPCR by the vendor of the reference standard (Fig. 5A).

In MRD detection and relapse monitoring, VAFs below 1% are common due to a low post-treatment tumour size. This requires high sensitivity and specificity during analysis, which is challenging due to the presence sequencing and PCR errors. To address this, GeneBits and umiVar were validated using cell-free DNA standards containing medium- to low-frequency variants.

Initially, the Horizon Std with dilutions of oncogenic variants (5%, 1%, 0.1%) and a wildtype control (0% VAF) was tested. For each sample, 10 ng (IDT workflow) or 30 ng (Twist workflow) of reference standard was used. Custom NGS panels were designed to target eight variants (six SNVs, two indels). All SNVs and one indel in the 5% and 1% standards were detected using both workflows, with median VAFs (4.8%/0.96% and 4.6%/0.88%) closely matching the expected VAFs (Fig. 5B). From the 0.1% standard, 5/8 variants were detectable but not “called” as they did not meet post-filter criteria (p-value ≤ 0.05, ≥ 3 consensus reads). The reduced sensitivity is likely due to limited coverage of Horizon Std samples (Fig. 4A and B), as a coverage of approximately 2000x after deduplication was insufficient to detect variants at 0.1% VAF. In the negative controls, all reads showed the wildtype sequences, indicating a 100% specificity under the chosen settings.

GeneBits was then applied to Twist Std v2, containing a set of dilutions of oncogenic variants (5%, 1%, 0.5%, 0.25%, 0.1%, 0% VAF) to represent patient samples with medium to very low tumour burden. From over 300 genomic alterations, 50 cancer-associated SNVs were selected for panel design. Using 30 ng of each reference standard, the IDT workflow detected all 50 variants in dilutions from 5 to 0.25% (Fig. 5C and D) post quality filtration. At 0.1% dilution, sensitivity still reached 94% (47/50 called). The calculated median VAFs (5.5%, 1.2%, 0.56%, 0.31%, 0.13%) matched the vendor’s predictions. In the negative control, no false positives were called, resulting in a specificity of 100%. Two variants showed a single consensus read with a false nucleotide change; however, single reads do not pass umiVar’s variant call quality filter.

The Twist workflow achieved 100% sensitivity down to the 0.25% standard and 82% for the 0.1% dilution, with calculated median VAFs 1.6-2 times higher than vendor’s specifications. No variants were detected in negative controls (100% specificity post quality filter).

### Benchmarking different DNA input amounts and probe tiling densities

The amount of cell-free DNA input is often limited to less than 30 ng due to insufficient plasma volumes or overall cell-free DNA low concentrations. To address this, the sensitivity and specificity were evaluated using only 10 ng of Twist Std v2 dilutions with the Twist targeted NGS workflow. Using a minimum UMI family size of four, the mean coverage at target sites was approximately 2.3-times lower for 10 ng input samples (739–1026 reads) compared to 30 ng samples (1707–2335 reads, Fig. 4B). However, error rates remained largely unaffected (Fig. 4D). All 50 variants were detected down to the 0.5% dilution (Fig. 4E). For the 0.25% dilution, the sensitivity was reduced to 80% (40/50 called), and dropped to 20% (10/50 called) for 0.1% dilution. No false positives were detected in the wildtype control (100% specificity). The reduced sensitivity is expected given the lower coverage and stringent post-filtering criteria. These data show that even with limited input, low-allelic frequency variants can be detected with high specificity.

Chromatin states in cells are highly dynamic, involving nucleosome repositioning to alter DNA accessibility [35]. This dynamic nature leads to variability in cell-free DNA fragment endpoints. We hypothesized that increasing the tiling density of hybridization capture probes might enhance enrichment efficiency by creating more precise overlaps between the probes and the targeted DNA. To test this, 120-bp probes with 3X tiling were designed for the IDT workflow and compared with the previous 2X tiling strategy (Fig. 3A). The capture reaction was performed using the identical pre-capture libraries of Horizon Std and Twist Std v2 dilutions to start with the identical library complexity. For Horizon Std, no major changes occurred with 3X tiling (Suppl. Figure 4 A and 4B). Again, 6/8 variants were called in 5% and 1% dilutions, while variants in 0.1% dilution were filtered out due to insufficient alternative reads. For Twist Std v2, the denser tiling also did not show a noticeable effect on enrichment efficiency and call accuracy. Sensitivity and specificity remained consistent with 2X tiling results (Fig. 5G and H). VAF variances were similar across dilutions, with one additional variant called in 0.1% dilution, increasing sensitivity from 94 to 96%. We conclude that 2X tiling is sufficient, while reducing the number of probes for the enrichment panel, allowing for more variants to be monitored.

### Benchmarking the new twist targeted NGS workflow

Twist recently released an updated version of their pre-capture workflow designed for low input cfDNA samples, aiming to increase library complexity and duplex read rates. Target enrichment was performed using the provided control panel covering all variants from Twist Std v2, including 224 SNVs. Despite using only 15 ng input DNA, the new workflow achieved a comparable number of mixed consensus reads to the previous 30 ng workflow (Suppl. Figure 3 A). Moreover, duplex read depth per nanogram was twice as high with the new kit (Supp. Figure 4C). The updated workflow showed sensitivity of 99% for dilutions down to 0.5%, 88% for the 0.25% dilution, and improved the detection rate from 20 to 39% at 0.1% dilution (Suppl. Figure 5 A and 5B). No false positives occurred in the 0% standard, indicating 100% specificity. These results demonstrate improvements in the new Twist workflow driven by increased library complexity and duplex read yield. Nonetheless, the IDT workflow provides the best detection rates at very low ctDNA levels (up to 96% call rate in the 0.1% dilution).

### Comparison of UmiVar with other pipelines

We compared umiVar’s performance with pipelines recommended by IDT and Twist, which use fgbio and Picard for UMI-assisted error correction and consensus sequence generation (Supp. Figure 3). All pipelines provide a duplex consensus (DC) mode. In alternative mode, IDT and Twist generate simplex consensus (SC), while umiVar generates mixed consensus (MC). The IDT pipeline utilizes VarDict for variant prediction. For benchmarking we used the 0.1% dilution of Twist Std v2, prepared with the IDT and Twist targeted NGS workflows.

In DC mode at minimum 4-fold UMI family size, comparable duplex read depths were achieved with all tested pipelines. Using the IDT library kit, both umiVar and IDT pipeline obtained a median of approximately 4000x DC read coverage (Suppl. Figure 6 A) with near-zero error rates. Using the Twist library kit, the umiVar and Twist pipelines generated approximately 1200x DC read coverage with error rates matching the performance of umiVar on the IDT library kit. In SC mode, Twist and IDT pipelines generate up to twice as many reads but with increased error rates (0.003–0.006%, Supp. Figure 6 C/D). UmiVar’s MC mode, which reports SC reads only if DC reads are not available for a DNA fragment, achieves a more balanced result.

The MC mode increases the yield by 44% (IDT) to 105% (Twist) compared to DC mode, while maintaining 3-4-times lower error rates (0.0007–0.002%, Supp. Figure 6 C/D) than SC mode of the competing tools. All tools predicted comparable VAFs (Suppl. Figure 6E and 6 F). A brief comparison between umiVar and VarDict shows slightly better sensitivity of umiVar on calling low-frequency variants (five variants called by umiVar are missed by VarDict, Supp. Figure 6 H), with otherwise highly correlated read coverage (*R* = 0.94) and VAFs (*R* = 0.93) between tools (Suppl. Figure 7 A and 7B). Benchmarking against the widely used VarScan2 variant caller also demonstrated similarly high agreement, with VAF and read coverage correlations of 0.98 and 0.95, respectively, on the same 0.1% reference standard (Suppl. Figure 7 C and 7D). Collectively, these findings further confirm the robustness and accuracy of umiVar’s variant calling at very low allele frequencies.

### Re-analysis: molecular residual disease detection in a longitudinal melanoma cohort

In the recent PET/LIT study [9], a GeneBits-like workflow was conceptually applied to monitor a melanoma cohort during therapy using liquid biopsy diagnostics alongside PET/CT imaging. A prior version of the umiVar bioinformatics pipeline was utilized for variant detection. We selected three patients from this study showing different responses to Ipilimumab/Nivolumab (Ipi/Nivo) treatment, validated by total lesion glycolysis (TLG) measurement. Tumour-informed panels targeting up to 30 SNVs were designed utilizing the panel design tool integrated in the clinical decision support system GSvar (Suppl. Figure 8 A). We reanalysed NGS data from cfDNA samples at minimum UMI family size of two, using the latest version of umiVar. Processed sequencing data were imported into GSvar for visualization and evaluation of quality metrics (Suppl. Figure 8B).

Patient A, whose imaging data indicated disease progression, exhibited an overall increase in VAFs between PET/CT scans (Fig. 1). Interestingly, during repeated drug administration, VAFs temporarily decreased by approximately 76%, indicating a strong treatment response. In contrast, ctDNA analysis for patient B demonstrated a strong response to Ipi/Nivo therapy. However, 78 days after treatment start, three variants remained detectable at significant levels, corroborated by a reduced TLG signal on PET/CT.

To assess the presence of MRD, the umiVar pipeline generates statistics based on variant versus wildtype reads at the combined target sites and their ± 60 bp flanks, followed by Fisher’s Exact test. For patients A and B, all timepoints indicated the presence of MRD, while patient C achieved complete response with no variants detected at the final timepoint. In additional exemplary cases, VAF kinetics again closely mirrored dynamics observed via PET/CT (Suppl. Figure 9). MRD detection was accompanied by a corresponding TLG signal in five of six cases, whereas MRD-negativity was followed by the absence of TLG signal in one case (patient F). These findings highlight the valuable clinical insights provided by the GeneBits approach.

## Discussion

In this study, we evaluated and benchmarked the GeneBits diagnostics workflow and the integrated umiVar software for monitoring somatic variants in plasma samples from cancer patients. The workflow was employed for panel design and NGS data analysis of three cfDNA reference standards. Read depths at target sites matched data published by IDT and Twist. VAFs calculated by umiVar correlated strongly with those predicted by reference material vendors. The old Twist workflow overestimated VAFs of Twist Std v2 by up to two-fold – a discrepancy noted by Twist in its validation experiments, published on the product homepage.

GeneBits and umiVar detect ultra-low frequency variants in cell-free DNA with high precision. The tumour-informed approach for small panel design enabled sequencing cfDNA samples at depths up to 100,000x - ten times higher than typical CAPP-seq experiments [27]. This ultra-high coverage enabled exceptionally low error rates, with a median of 0.00036% for Twist Std v2 libraries in DC mode, lower than shown on the Twist product page (~ 0.001%), and comparable to iDES-enhanced CAPP-seq (0.0004–0.0072%) [36]. With LODs as low as 0.0017% in DC mode, GeneBits shows better sensitivity than other tumour-informed methods such as iDES-enhanced CAPP-seq (~ 0.004% VAF) and Invitae Personalized Cancer Monitoring assay (~ 0.008%), and similar sensitivity as the RaDaR assay (0.0011%) [17, 32, 37]. On average, duplex calling reduced error rates 2.7-fold versus mixed consensus, though mixed consensus offers a practical balance between target coverage depth and error rate. When sufficient input DNA is available or coverage depth is not a limiting factor, filtering for duplex reads may be preferred.

For both targeted NGS workflows, the pipeline detected most variants in Twist Std v2 with 10 ng input DNA at VAFs as low as 0.1%, while maintaining error rates over two orders of magnitudes lower. UmiVar’s stringent filters for variant calling achieved 100% specificity in negative controls, a critical requirement in clinical settings to avoid false-positive ctDNA detection.

For the 0.1% Horizon Std, consensus read depths were below 3000x - the minimum to detect three variant copies in 10 ng cfDNA. Therefore, we recommend using 20–50 ng input DNA for higher library complexity. This amount has also been recommended by other studies for low-frequency variant detection and corresponds to the average yield of cfDNA isolated from 2 to 4 ml plasma from patients with stage I-III cancer [38–40]. Higher input also reduces replicate-dependent variability in calculated allele frequencies caused by Poisson sampling errors [39, 41]. However, lower inputs may still be used when material is limited, though this results in reduced coverage and, consequently, lower detection sensitivity.

Expanding the size of the NGS panel may enhance variant detection sensitivity and improve statistical power by increasing the number of informative sites. Previous studies have shown a positive correlation between the number of monitored SNVs and sensitivity, with recommended panel sizes typically ranging from 10 to 70 SNVs. Beyond this range, additional gains tend to plateau [32, 42]. In GeneBits, using the Twist platform, we achieved a sensitivity of 20% in the 0.1% dilution of Twist Std v2. Based on these findings, we recommend a panel size between 20 and 100 SNVs. At the lower end, this enables detection of approximately five variants, which is sufficient to meet our minimum threshold of three variants required to call MRD positivity. The upper end aims to maximize sensitivity while avoiding unnecessary redundancy.

We also tested the effect of probe density (tiling) at the target sites and found no noticeable difference in performance between 2X and 3X tiling. With 2X tiling, the probability that at least one of the two probes captures the variant is approx. 96–98%, making the added redundancy of 3X tiling unnecessary. In contrast, 1X tiling may lead to loss of SNVs due to variability in oligo efficiency. Therefore, we propose that 2X tiling offers the best balance between sensitivity and cost efficiency.

The umiVar pipeline was validated against comparable workflows, and results closely aligned the pipelines adopted by IDT and Twist for generating consensus reads. Unlike IDT’s workflow, the umiVar barcode correction tool does not require a UMI reference sequence file, making it compatible with fixed (IDT) or randomized (Twist) UMI designs. The umiVar variant caller showed highly sensitive detection of low-frequency variants, outperforming VarDict for variants with 0.1% VAF. In addition, umiVar generates statistics and plots for quality metrics, including error rates, nucleotide-specific error profiles, read coverage by duplication level, MRD statistics, and VAF metrics, which are not provided in the IDT and Twist pipelines.

In clinical application, GeneBits provided valuable information on therapy response. VAF kinetics aligned with PET/CT imaging results [9]. UmiVar identified the presence of molecular residual disease in seven patients and its absence in two patients by differentiating true variants from PCR- and sequencing-associated background noise. Assessing the prognostic value of MRD detection was beyond the scope of this study, which focussed on benchmarking the technical aspects of the workflow. However, previous studies have demonstrated the utility of MRD in predicting disease recurrence and overall survival, underscoring the clinical relevance of developing highly-sensitive tools for MRD detection [13–18].

With turnaround times of less than a month for baseline samples and two weeks for subsequent timepoints, ctDNA analysis can inform clinical decisions in timely manner. The processing timeframe has previously proven to be clinically applicable, thus offering a relatively fast and continuous assessment of tumour burden compared to imaging, the latter being recommended only every 3–6 months during adjuvant therapy or 6–12 months during follow-up [43–45].

We acknowledge that translating GeneBits into clinical practice poses logistical challenges, including timely tumour-normal sequencing, serial plasma collection at regular intervals and storage in a −80 °C freezer. Moreover, a customized panel design rather than standardized cancer gene panels is required, which requires automation to facilitate scalability. NGS data must be processed, analysed by authorized personnel, and reported to clinicians. As a result, the entire diagnostic process involves multiple steps and various professional roles. Standard operating procedures (SOPs) are essential to ensure consistency, streamline workflows, and enable a sufficiently short turnaround time (TAT). The use of specialized collection tubes for cfDNA (e.g., Streck Cell-Free DNA BCT, PAXgene Blood ccfDNA tube) allows temporary storage of blood at room temperature for up to 7 days after collection, thereby easing time constraints and providing greater flexibility in sample processing workflows. The integration of personalised panel design into a clinical decision support system like GSvar can help streamline workflows and enable seamless data sharing across laboratory and clinical teams.

Additionally, a tumour-informed approach cannot effectively detect resistance mutations or clonal haematopoiesis mutations emerging during therapy [46–48]. This limitation can be addressed by supplementing the tumour-informed panel with a predefined set of resistance-associated hotspots or entire resistance genes, achieved either through adding an extra probe pool during hybrid capture or by incorporating both components into a single panel design from the outset. However, this approach would require deeper sequencing to maintain the very high coverage necessary for sensitive detection.

In this study we did not benchmark the performance of umiVar for detecting unknown resistance mutations, which would require an increased region of interest, unless one focusses on a limited number of known resistance hotspots. This leads to a higher probability of false positives, which can be compensated by increasing the LOD through reduced *p*-value thresholds, for the price of reduced sensitivity. However, this needs to be benchmarked in a separate benchmark test using specialised reference standards or patient samples with known resistance mutations, which was beyond the scope of this study.

A further challenge in MRD detection is posed by overall low ctDNA shedding rates, which can vary depending on tumour type and stage, or by restricted vascular access of the tumour tissue [10, 49, 50]. In such scenarios, obtaining greater plasma volumes is necessary to yield sufficient cfDNA input, accompanied by increased sequencing depth. For these cancer types, MRD predictions must be interpreted with greater caution, and alternative diagnostic approaches may be necessary. Future prospective studies will be required to systematically evaluate the clinical performance of MRD detection across different tumour entities, disease stages, and patient populations.

## Conclusion

The GeneBits workflows outlines the collection and processing of liquid biopsy samples for tumour-informed variant monitoring during cancer treatment. A key component is the open-source software umiVar, providing error correction, variant calling, MRD detection, and longitudinal analysis of treatment outcome. GeneBits was rigorously benchmarked using targeted NGS workflows from IDT and Twist, with three commercial cell-free DNA reference standards as input. Mixed consensus calling in umiVar achieved exceptionally high sensitivity for low-frequency variants while maintaining 100% specificity in wild-type controls. Clinical validation in patient samples accurately predicted the presence of molecular residual disease, as confirmed by PET/CT imaging. We propose integrating GeneBits as a monitoring tool in clinical studies and routine diagnostics to support decision-making.

## Supplementary Information

Supplementary Material 1

Supplementary Material 2

Supplementary Material 3

## Data Availability

All raw sequencing data from cfDNA reference standards will be made available on the Sequence Read Archive (SRA) under the following BioProject ID: PRJNA1219499. Raw sequencing data from melanoma patients were obtained from Schroeder et al. 2024.

## Abbreviations

AF: Allele frequency
BAM: Binary alignment map
bp: Base pair
cfDNA: Cell-free DNA
ctDNA: Circulating tumour DNA
DC: Duplex consensus
ddPCR: Droplet digital PCR
Ipi/Nivo: Ipilimumab/Nivolumab
LB: Liquid biopsy
LOD: Limit of detection
MC: Mixed consensus
MRD: Molecular residual disease
ng: Nanogram
NGS: Next-Generation Sequencing
PET/CT: Positron emission tomography/computed tomography
SC: Simplex consensus
SNP: Single-nucleotide polymorphism
SNV: Single-nucleotide variant
Std: (Reference) standard
TAT: Turnaround time
TLG: Total lesion glycolysis
UMI: Unique molecular identifier
VAF: Variant allele frequency
WES: Whole-exome sequencing
