# Supplementary material for: GeneBits: ultra-sensitive tumour-informed ctDNA monitoring of treatment response and relapse in cancer patients
Source: J Transl Med. 2025 Aug 27;23:964. doi: 10.1186/s12967-025-06993-3 (PMC12382282; doi:10.1186/s12967-025-06993-3)
Supplement: Supplementary file 1 — Supplementary Material 1 [file 12967_2025_6993_MOESM1_ESM.docx]

# Ultra-sensitive tumour-informed ctDNA monitoring of treatment response and relapse in cancer patients with GeneBits

Julian Broche, Olga Kelemen, Aishwarya Sekar, Leon Schütz, Francesc Muyas, Andrea Forschner, Christopher Schroeder, Stephan Ossowski

# SUPPLEMENTAL MATERIALS

## Supplemental Figures

Supplementary Figure 1: Time course of the GeneBits workflow for somatic variant monitoring in plasma samples.

Supplementary Figure 2: Comparison of bioinformatics pipelines for deduplication and variant detection.

Supplementary Figure 3: Yield of deduplicated and duplex reads for cell-free DNA reference standards calculated with umiVar.

Supplementary Figure 4: Probe tiling density does not improve target enrichment efficiency in the Horizon standard.

Supplementary Figure 5: Sensitive detection of low-frequency variants with the new Twist targeted NGS workflow.

Supplementary Figure 6: Performance of error correction and consensus sequence generation of umiVar compared to computational pipelines recommended by IDT and Twist.

Supplementary Figure 7: Comparison of umiVar variant caller with VarDict and VarScan2.

Supplementary Figure 8: Integration of tumour-informed panel design tool and umiVar variant calling results into the clinical decision support system GSvar.

Supplementary Figure 9: Additional melanoma cases monitored during treatment using the GeneBits workflow.

Supplementary Information: UmiVar bioinformatics pipeline - barcode correction, variant calling and MRD detection.


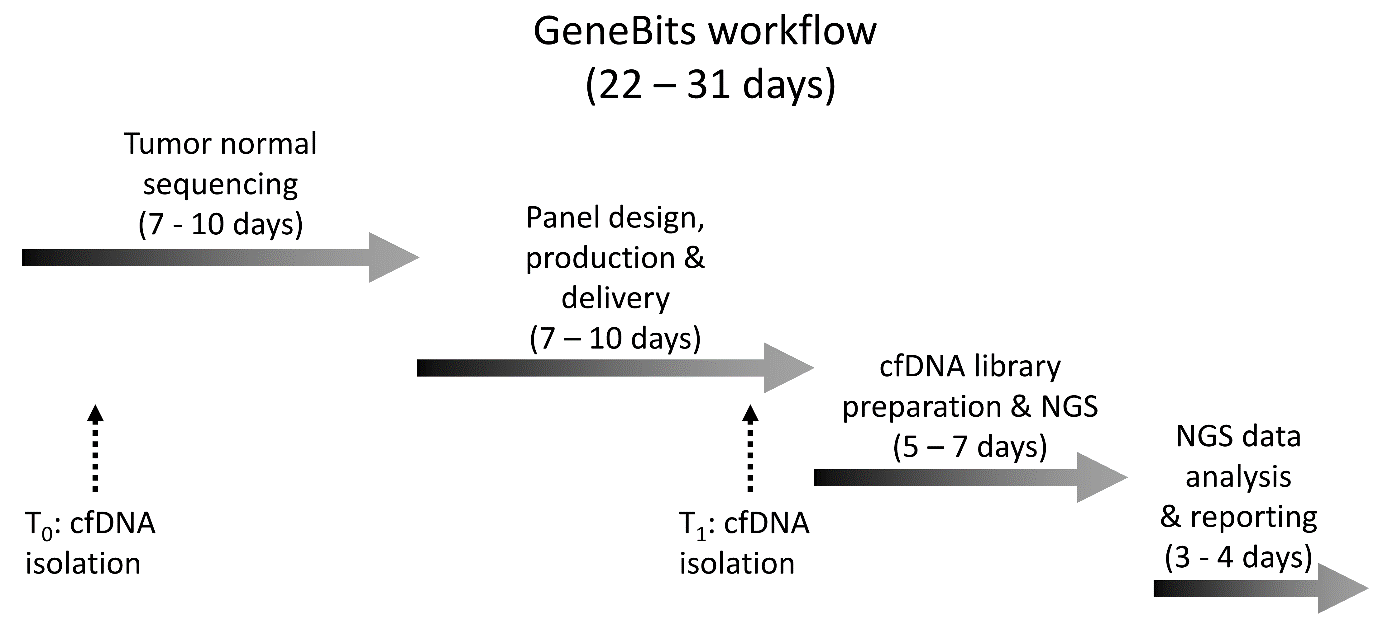


**Suppl. Figure 1: Time course of the GeneBits workflow for somatic variant monitoring in plasma samples.** Cell-free DNA (cfDNA) is isolated upon arrival and targeted Next-Generation Sequencing (NGS) is performed with the baseline (T_0_) sample and, optionally, the first treatment sample (T_1_), if available. Estimated length for processing steps shown in brackets.


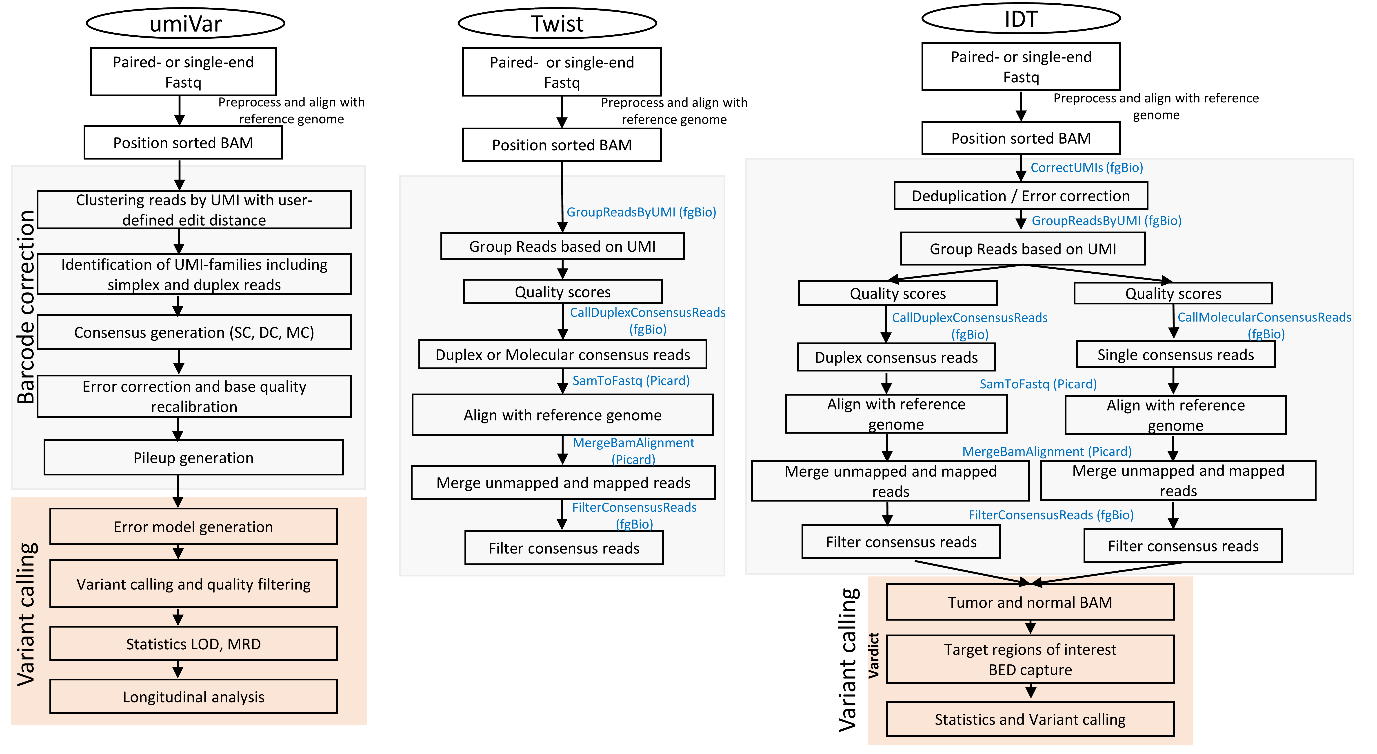


**Suppl. Figure 2: Comparison of bioinformatics pipelines for deduplication and variant detection.** All processing steps in umiVar and the pipeline proposed by IDT and Twist are displayed. Processing steps for unique molecular identifier (UMI)-based error correction are highlighted in gray, and for variant calling in orange. Twist and IDT pipelines both utilize tools from the Fgbio (<http://fulcrumgenomics.github.io/fgbio>) and Picard (<https://broadinstitute.github.io/picard>) toolkits.


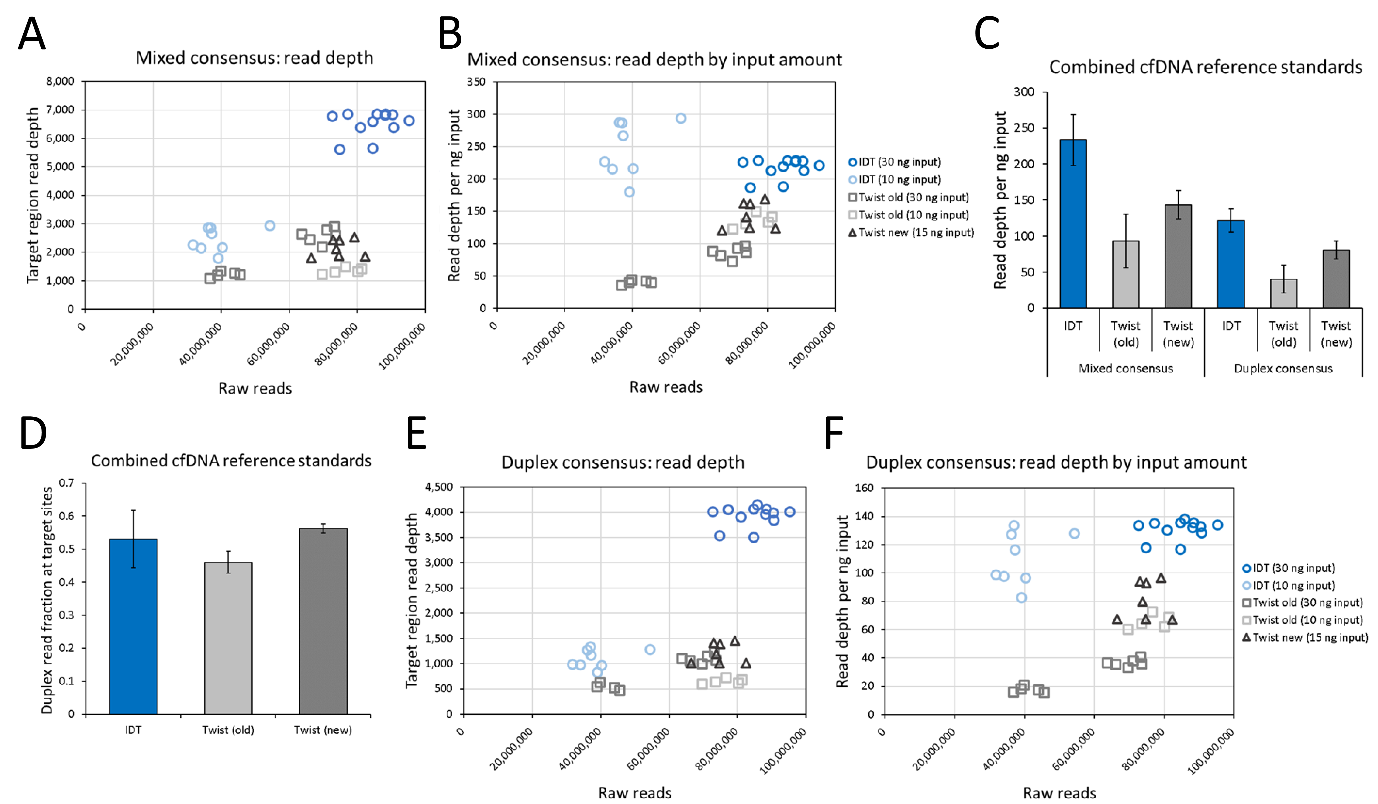


**Suppl. Figure 3: Yield of deduplicated and duplex reads for cell-free DNA reference standards calculated with umiVar.** Read depths calculated at ≥1x UMI family size. **(A)** Mean target region read depths of mixed consensus (MC) reads at target sites for ≥1 UMI-family size, compared to raw sequencing input. **(B)** Mean target region read depths, normalized to input amount and compared to raw sequencing input. **(C)** Aggregated statistics of mean target region read depths, normalized by input amounts, shown for IDT, Twist (old) and Twist (new) targeted NGS workflows in mixed or duplex consensus mode. **(D)** Duplex read fraction of combined cfDNA reference standards at target sites for different targeted NGS workflows. **(E)** Mean duplex read depths of cfDNA reference standards compared to raw sequencing input. **(F)** Mean duplex read depth normalized to cfDNA input amount and compared to raw sequencing input.


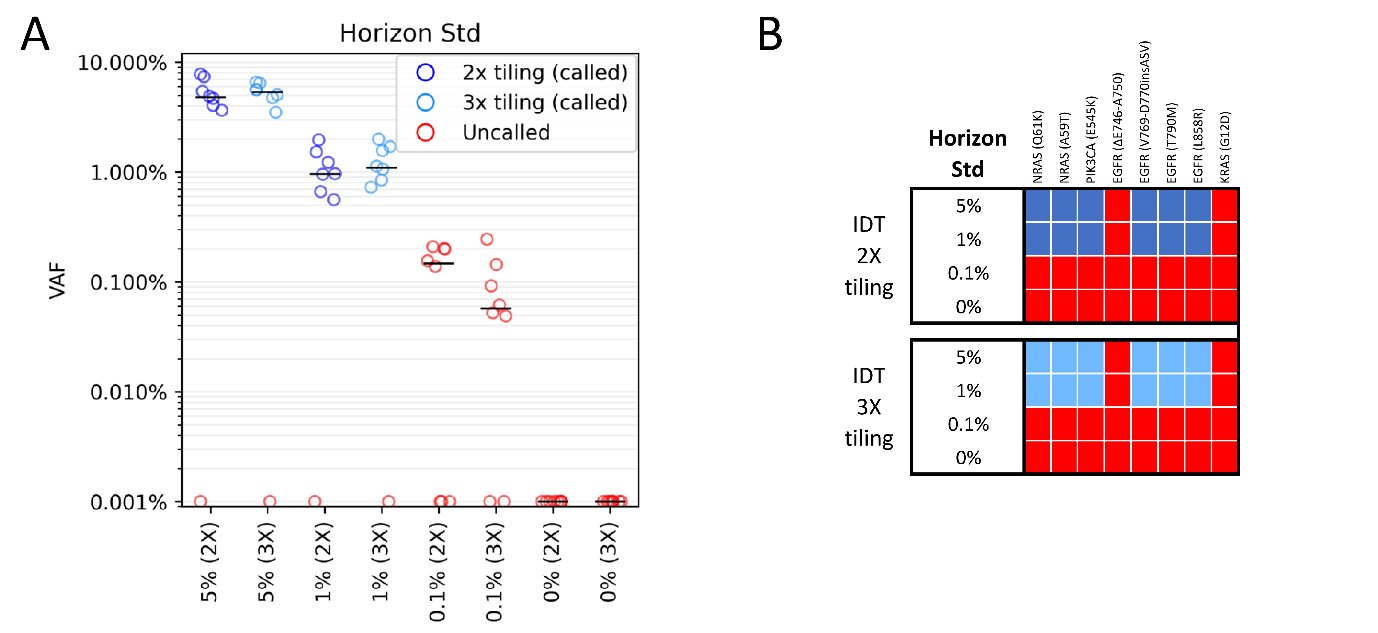


**Suppl. Figure 4: Probe tiling density does not improve target enrichment efficiency in the Horizon standard. (A)** Calculated variant allele frequencies (VAF) of variants included in the Horizon Std. Target enrichment was performed with 2x and 3x probe tiling density. Pseudocount of 0.001% added to all variants. **(B)** Display of panel-wide variants by Horizon Std dilution levels. A) and B) Blue = variant called, red = uncalled (< 3 variant reads, and/or p-value > 0.05).

**
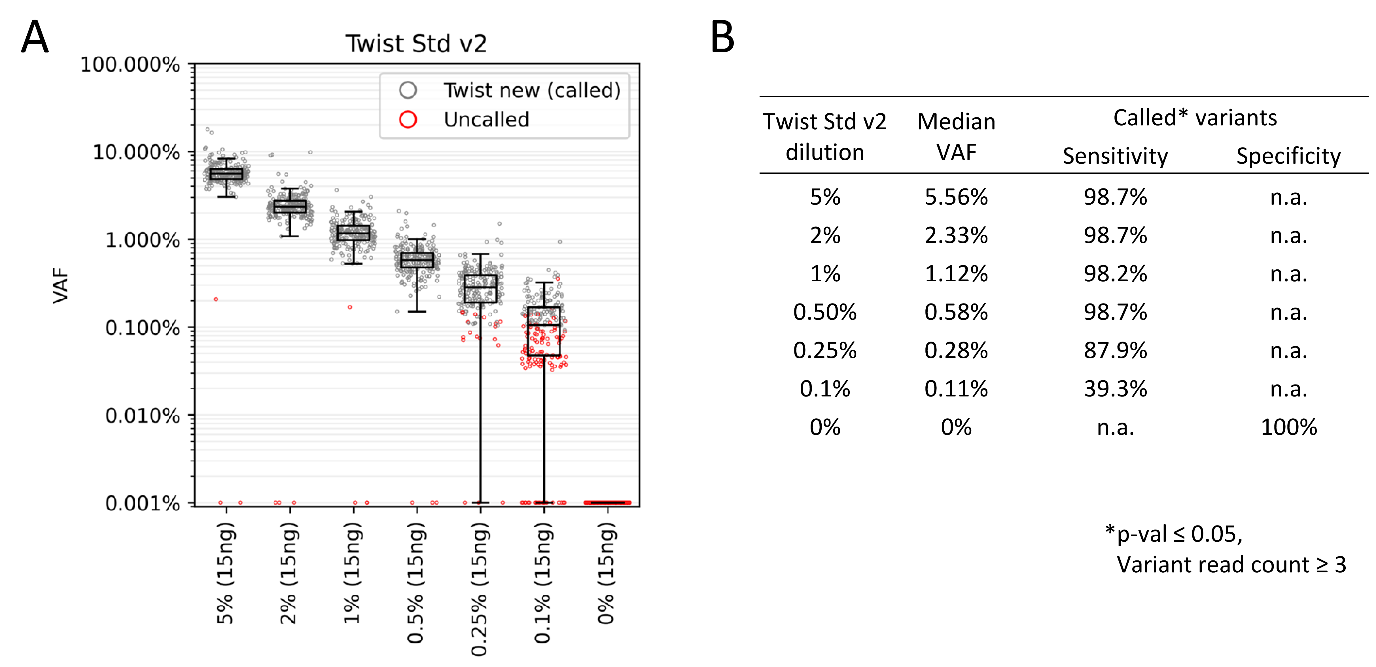
**

**Suppl. Figure 5: Sensitive detection of low-frequency variants with the new Twist targeted NGS workflow.** Benchmarking of the workflow with all available Twist Std v2 dilutions using 15 ng input. **(A)** Calculated variant allele frequencies (VAF) for the 5% to 0% dilutions. All single-nucleotide variants (SNVs) present in the reference material were included into the enrichment panel. Pseudocount of 0.001% added to all variants. Gray = variant called, red = uncalled (< 3 variant reads, and/or p-value > 0.05). **(B)** Sensitivity (5% - 0.1% dilution) and specificity (0% control) of the new Twist workflow for Twist Std v2, only considering called variants.


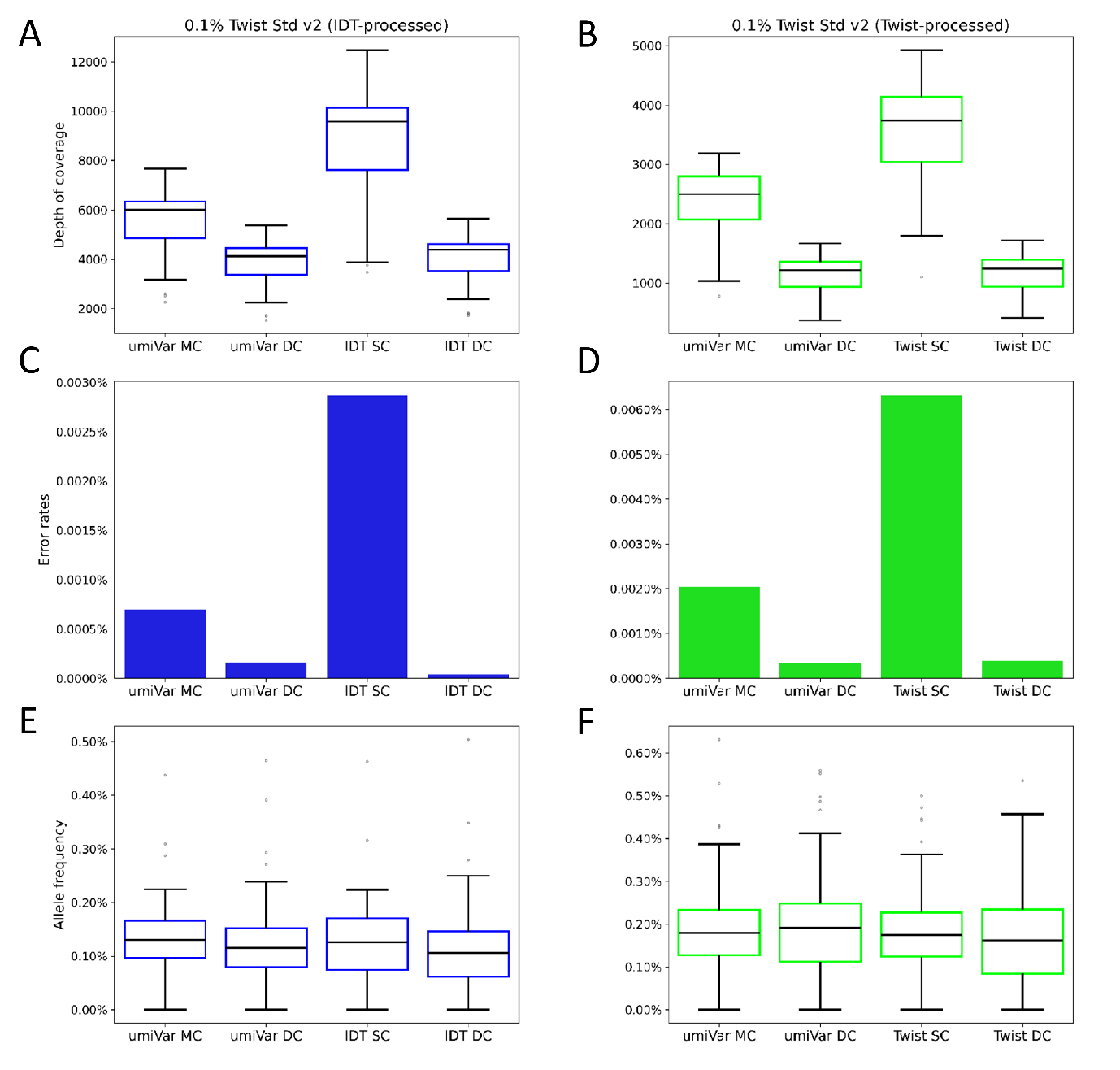


**Suppl. Figure 6: Performance of error correction and consensus sequence generation of umiVar compared to computational pipelines recommended by IDT and Twist.** Targeted NGS workflows from IDT and Twist were used for the 0.1% dilution sample from Twist Std v2. IDT libraries (blue) were analysed with the IDT bioinformatics pipeline, Twist libraries (green) with the Twist bioinformatics pipeline and compared with umiVar for ≥4 UMI-family size. SC = simplex consensus, DC = duplex consensus, MC = mixed (SC/DC) consensus. **(A)** Target region read depths for the umiVar- and IDT-processed IDT library. **(B)** Target region read depths for umiVar- and Twist-processed Twist library. **(C)** Error rates for the umiVar- and IDT-processed IDT library. **(D)** Error rates for the umiVar- and Twist-processed Twist library. **(E)** Variant allele frequencies for umiVar- and IDT-processed IDT library (0.1% expected VAF). **(F)** Variant allele frequencies for umiVar- and Twist-processed Twist library (0.1% expected VAF).


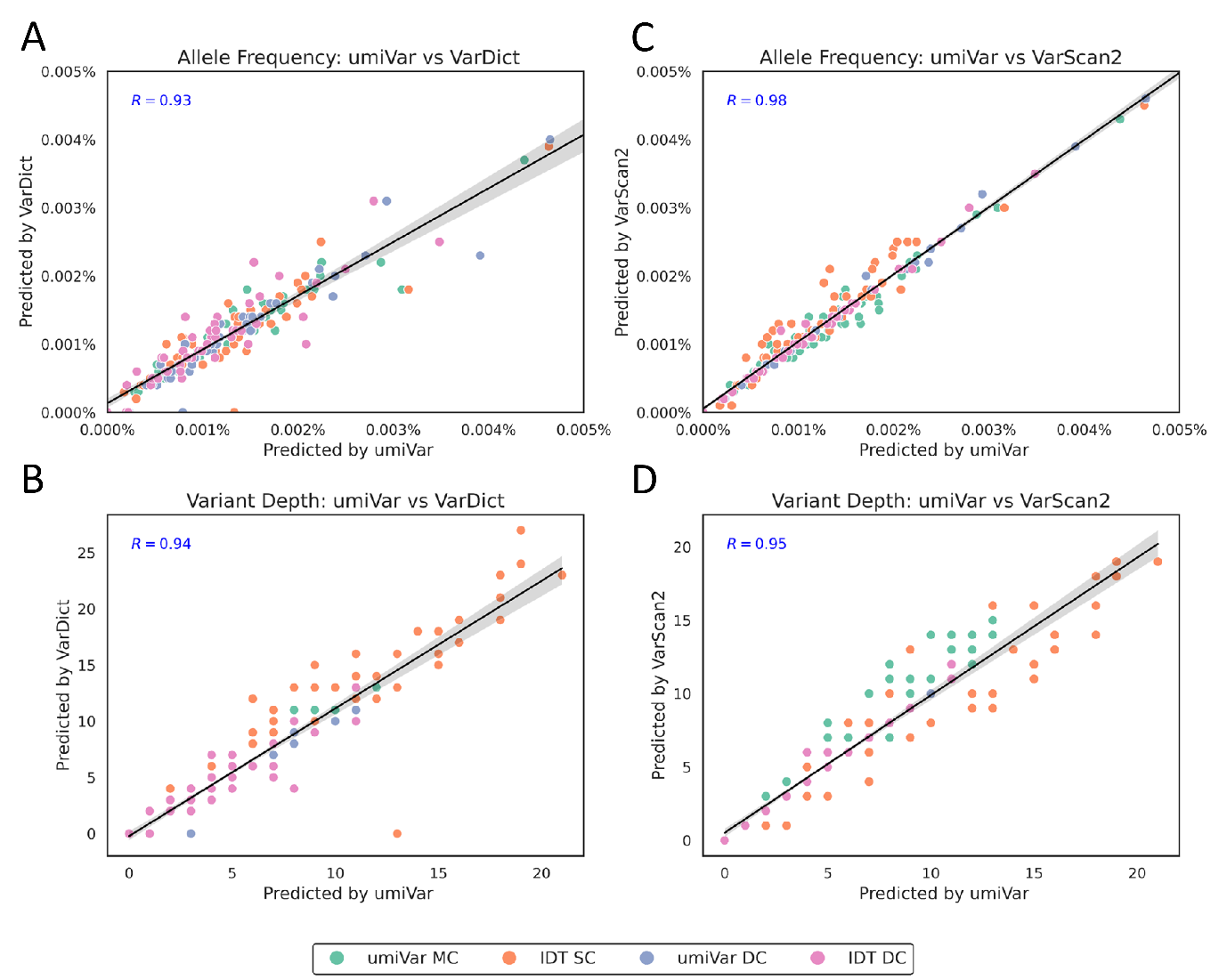


**Suppl. Figure 7: Comparison of umiVar variant caller with VarDict and VarScan2.** The targeted NGS workflow from IDT was used for 0.1% dilution sample from Twist Std v2. 50 SNVs were included into the panel. The library was analysed with the IDT bioinformatics pipeline and compared with umiVar for ≥4 UMI-family size. SC = simplex consensus, DC = duplex consensus, MC = mixed (SC/DC) consensus. *R* = Coefficient of multiple correlation. **(A)** Variant allele frequencies called by umiVar (x-axis) vs. VarDict (y-axis). **(B)** Variant depths called by umiVar vs. VarDict. **(C)** Variant allele frequencies called by umiVar vs. VarScan2. **(D)** Variant depths called by umiVar vs. VarScan2.


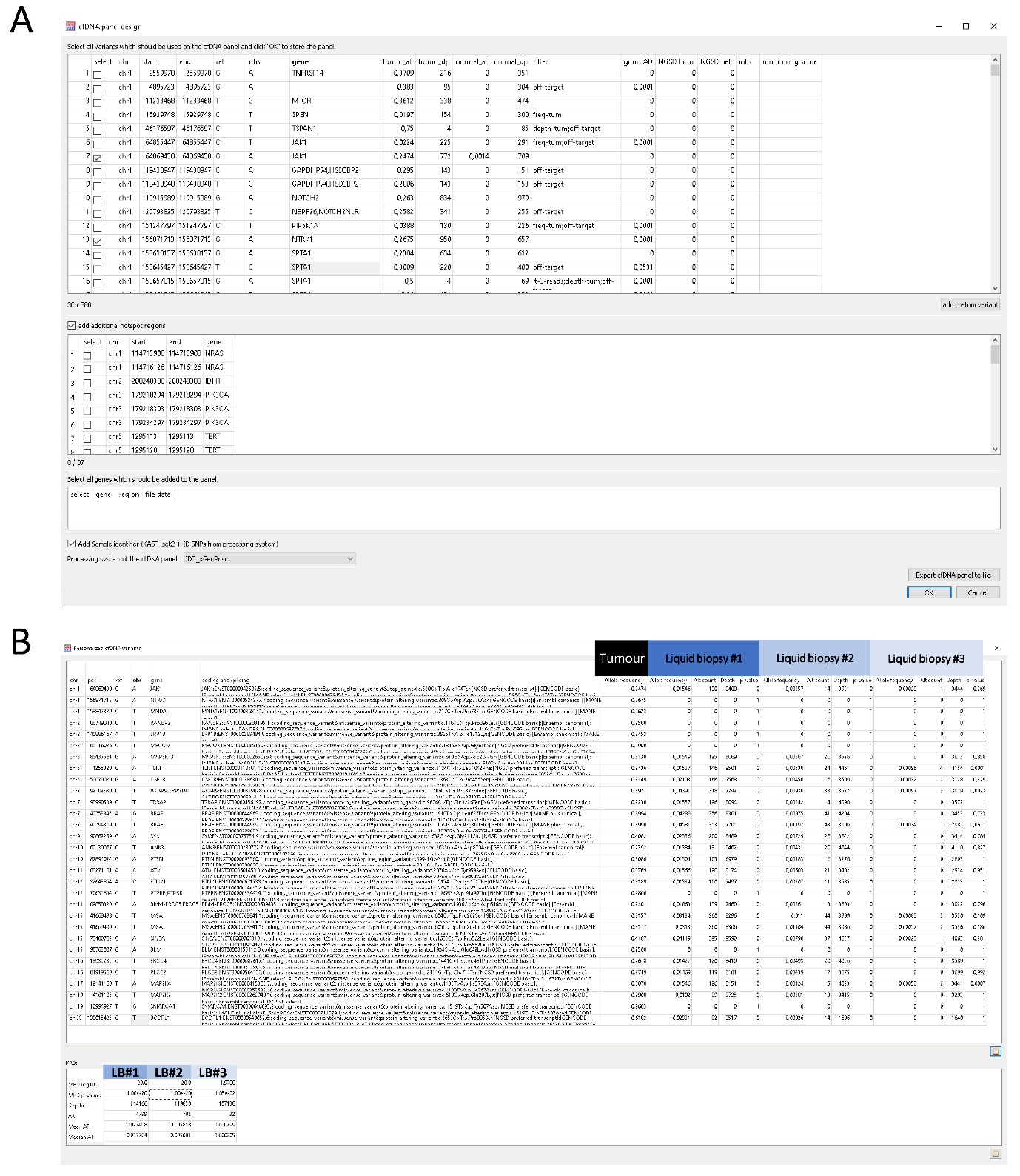


**Suppl. Figure 8: Integration of tumour-informed panel design tool and umiVar variant calling results into the clinical decision support system GSvar.** **(A)** Panel design tool for the selection of somatic variants, additional hotspot region and the employed NGS workflow. **(B)** Example of an umiVar variant calling output for a patient with three liquid biopsies. Variant allele frequencies, alternative counts (“Alt_count”), target region read depth (“Depth”) and p-values are shown for each sample timepoint. Bottom: residual disease statistics for each liquid biopsy (LB) tested.


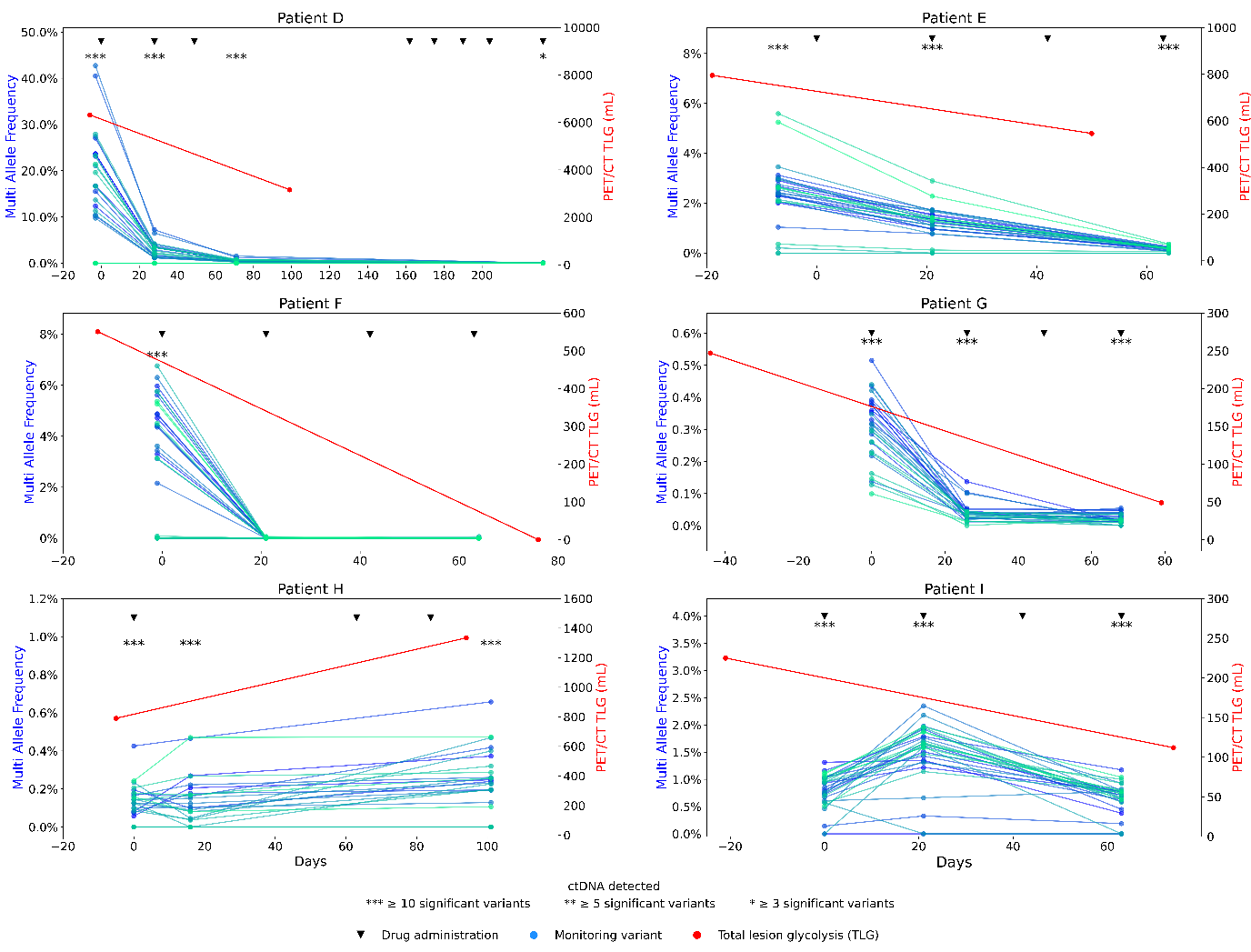


**Suppl. Figure 9: Additional melanoma cases monitored during treatment using the GeneBits workflow.** Data obtained from PET/LIT study (PMID: 39384805). Reanalysis of six patients receiving Ipilimumab / Nivolumab treatment (▼) with the latest version of umiVar. A tumour-informed panel containing 30 variants was designed for each patient. Variant allele frequencies (VAF) for all variants were calculated with umiVar for ≥ 2x UMI-families. VAF kinetics are displayed for each of the monitored variants (blue/turquoise dots). Total lesion glycolysis (TLG, red dots) was measured by PET/CT before the start (Day 0) and around the end of therapy. Asterisks indicate the number of significant (Fisher’s Exact test) variants per liquid biopsy. ctDNA = circulating tumour DNA.

**Supplementary Information**

UmiVar bioinformatics pipeline - barcode correction, variant calling and MRD detection

The main goal of umiVar is to correct FFPE-, PCR- and sequencing-related errors by grouping the reads based on similarity of UMI sequences and collapsing them to preserve true variants.

The reads are grouped based on fragment endpoints and UMIs.

Input:

- Barcode correction
- Aligned BAM file
- Barcodes – start position | end position | both start and stop
- Maximum number of sequencing errors in grouping barcodes – threshold
- Flag n – to use N as the base instead of reducing base quality to zero

umiVar variant calling

- Tumor BAM file | normal BAM file
- Target BED file
- Reference genome (as FASTA)

Output:

- Mixed consensus BAM file
- Duplex consensus BAM file
- Variant calling TSV files
- MRD prediction
- QC metrics, pile-up files

Procedure:

1. UMI aware deduplication
   1. PCR duplicates are grouped based on unique molecular identifiers (UMIs)
   2. Extract UMI from every read based on start, end or both positions
   3. Group reads by fragment endpoints, UMI sequence similarity and orientation
   4. Allow a tolerable edit distance of mismatches in barcodes (recommendation: 1-3 mismatches, based on combined UMI length)
2. Consensus read calling
   1. Apply majority voting for every base based on the multiple sequence alignment score
   2. Mask the bases with ‘N’, where there is <75% agreement
   3. UMI families with lesser mean base quality than the threshold and too many discordant positions are removed
   4. Generate consensus BAM files. The reads are tagged with YD for duplex consensus (duplex: YD = 1; simplex: YD = 0). The BAM files are color coded based on duplication levels (‘YC’ tag) to allow duplication-based visualization in IGV
3. Error modeling
   1. Beta-binomial error model is computed for each type of nucleotide change type A>C, A>G, A>T, C>A, C>G, C>T
   2. Error models are also calculated based on UMI family size (1x, 2x, 3x and ≥4)
   3. Error models for autosomes are computed to filter common variants and variants with AF>20%
4. Variant calling
   1. At each position, observed allele frequency, background AF from error model, Fisher’s exact test, Z score, limit of detection are computed
   2. True variant is called if present in ≥ 3 different consensus reads and at a p value ≤0.05
5. Tumour-informed MRD detection
   1. Outlier removal: monitored variants with VAF > ±3 standard deviations from mean are filtered out.
   2. Collect all the mutant and wildtype reads from the target sites and compare to ±60bp flanks
   3. MRD significance is assessed by Fisher’s Exact test

Flowchart: bioinformatics pipeline


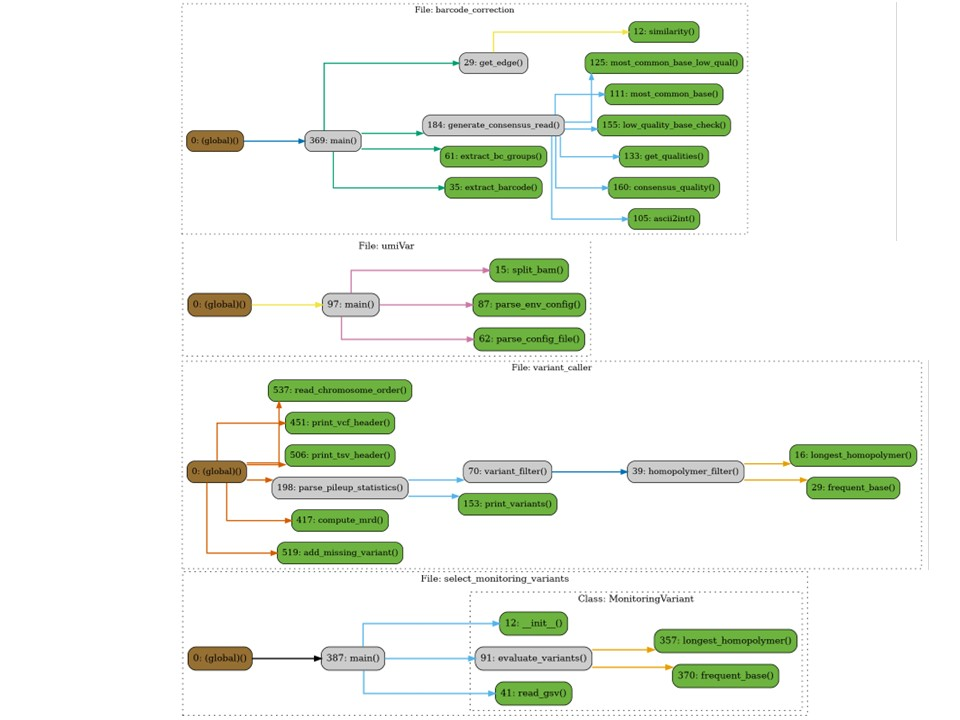


UmiVar code availability

Barcode correction: <https://github.com/imgag/umiVar/blob/main/barcode_correction.py>

UmiVar: <https://github.com/imgag/umiVar/blob/main/umiVar.py>

Variant caller: <https://github.com/imgag/umiVar/blob/main/variant_caller.py>

MRD calculation: <https://github.com/imgag/umiVar/blob/main/calculateMRD.py>

Pseudocode

FOR each read IN BAM_file:

EXTRACT UMI from start, end, or both positions

RECORD fragment start, end, orientation, and UMI

GROUP reads BY:

- fragment start and end positions

- read orientation

- UMI similarity (allow edit distance ≤ 3)

FOR each group:

CREATE UMI family

FOR each UMI family:

ALIGN all reads using multiple sequence alignment

FOR each base position:

COUNT base occurrences

IF majority base frequency ≥ 75%:

CALL consensus base

ELSE:

MASK base as 'N'

IF mean base quality < threshold OR too many discordant bases:

DISCARD UMI family

ELSE:

GENERATE consensus read with Tags:

YD = 1 if duplex

YD = 0 if simplex

YC = duplication level

OUTPUT all consensus reads to BAM file

OUTPUT duplex consensus reads to a BAM file

FOR each substitution type [A>C, A>G, A>T, C>A, C>G, C>T]:

FOR each duplication level [1x, 2x, 3x, ≥4x]:

COMPUTE background beta-binomial error model

FILTER:

- germline variants

- remove common variants

- exclude variants with AF > 20%

FOR each position in target BED regions:

CALCULATE:

- observed allele frequency (AF_obs)

- expected background AF from error model (AF_exp)

- Fisher’s exact p-value

- Z-score

- Limit of Detection (LOD)

IF variant present in ≥3 consensus reads AND p < 0.05:

CALL as true variant

FOR each variant in patient-specific list:

IF VAF > mean ± 3 * SD:

FILTER variant

FOR each remaining variant:

COLLECT:

- mutant and wild-type reads

- reads from ±60bp flanking regions

APPLY Fisher’s Exact test between mutant and flanking regions

IF p < significance threshold:

MARK as MRD positive
